# Integrative analysis of transcriptome and metabolome reveals molecular mechanisms of dynamic change of storage substances during dehydration and drying process in peanuts (*Arachis hypogaea* L.)

**DOI:** 10.3389/fpls.2025.1567059

**Published:** 2025-04-16

**Authors:** Jingjing Deng, Mingyu Hou, Shunli Cui, Yingru Liu, Xiukun Li, Lifeng Liu

**Affiliations:** State Key Laboratory of North China Crop Improvement and Regulation, Key Laboratory for Crop Germplasm Resources of Hebei, North China Key Laboratory for Crop Germplasm Resources of Education Ministry, Hebei Agricultural University, Baoding, China

**Keywords:** peanut, drying process, storage substance, transcriptome, metabolome

## Abstract

Various substances in seeds occurred many transformations during the drying process, which is key to long-term storage, but the mechanism is unclear. In this study, seeds of the peanut (*Arachis hypogaea* L.) variety “Silihong” were selected as the experimental materials. Transcriptome and metabolome analyses of the peanut kernels at day 0 (S0d), day 1 (S1d), day 3 (S3d), day 5 (S5d), and day 7 (S7d) of drying were performed to search for the genes that controlled the storage compounds. A total of 165 differentially expressed metabolites (DAMs) and 15,010 differentially expressed genes (DEGs) in the five stages of seed drying were identified. S3d was the key period during which the content of most of the metabolites changed significantly. The contents of most amino acids(87%) and their derivatives decreased significantly, and most of the lipids(68%), sugars(67%) and flavonoids(87%) accumulated to their peak at S3d. A Kyoto Encyclopedia of Genes and Genomes (KEGG) pathway analysis revealed that the DEGs were primarily enriched in four aspects, including amino acid biosynthesis and metabolism, lipid biosynthesis and metabolism, sucrose and starch metabolism, and flavonoid biosynthesis. Crucial genes that potentially regulate the storage substances were identified, including *PAL*, *FAD2*, *SUS*, *LOX*, and *PFK*. Overall, this study provides valuable insights into the molecular regulation of storage compounds in peanut seeds and may help to assess edible peanuts that have enhanced nutritional and economic values.

## 1 Introduction

Starch, protein, and lipids are important storage components in crop seeds, and the main types of storage substances vary depending on the type of crop. Oil are the main storage substances in cultivated peanut (*Arachis hypogaea*. L) which is important cash crop (Zhang et al., 2018). Moreover, the content and proportion of these primary metabolites are important indexes to evaluate the quality and determine the specialization direction of crop seeds. The protein content of special flour for bread is more than 13.5%, while the protein content of special flour such as steamed bread and noodles is between 12% and 13.5%. The peanut with oleic acid content more than 75% is classified as high oleic acid peanut, and sucrose content in fresh peanut is more than 6% (Talcott et al., 2005; Pattee et al., 1998). The content and proportion of various storage components are closely related to the maturity and drying status of the seeds.

There are significant changes in nutrients during the development of peanut seeds. During the development of peanut kernels, the contents of oil and protein increase gradually. The oil content increases from 41.84% in the yellow 1 stage to 49.95% in the black stage, and the protein content increases from 22.20% in the yellow 1 period to 24.10% in the black period (Wang et al., 2018). As the raw material for the biosynthesis of other substances, the trend of changes in the content of sucrose during the development of the seed kernels was opposite to that of protein and oil. The content of sucrose gradually decreased as the seeds developed. The sucrose content of peanut variety “ICG12625” was 168.1 mg/g at 15 days after flowering and 40.81 mg/g at 70 days after flowering (Li et al., 2021). The composition of peanut seeds undergoes tremendous changes from fertilization to maturity. In addition, the transcriptome also revealed that the biosynthesis of protein, oil and sugar during the development of seeds primarily occurred between the growth of seeds and the expansion of immature seeds (Gupta et al., 2016).

The seeds contain approximately 30%-40% of water after they reach maturity (Payman et al., 2011). Drying and dehydration is an important processing link after the seeds have been harvested. They dehydrate when mature to germinate and become tolerant to dehydration. With the increase in drying days, the water in the seeds gradually decreases, and the free amino acids, free fatty acids and carbohydrates are gradually transformed into proteins, fats and starches, which are stored in the seeds. The contents of soluble sugar and soluble protein in wheat (*Triticum aestivum* L.) increase after drying, and the contents of crude protein and oil in green algae increase after drying (Chen L. et al., 2018; Uribe et al., 2019). Peanut seeds contain approximately 50% oil. Compared with the seeds of wheat and rice (*Oryza sativa* L.) that have a high content of starch and those of soybean (*Glycine max* L.) with a high content of protein, the metabolism during drying also varies a great deal. However, during the drying process, the changes in the seed storage substances and their transcriptional regulatory mechanisms remain unclear.

Transcriptome and metabolome analyses are indispensable for investigating gene expression patterns, metabolic pathways, and regulatory mechanisms within living organisms. A comprehensive integration of these two analytical approaches has emerged as a pivotal methodology for elucidating the intricate relationship between genes and metabolites, thereby facilitating a deeper understanding of complex biological traits. In crop studies, the method revealed the effects of sweet potato (*Ipomoea batatas* (L.) Lam.) Patterns of anthocyanin, starch, and carotenoid accumulation during tuber development and provide important insights into the adaptation of Chieh-qua to *Fusarium oxysporum* infection (Lin et al., 2025; Qiao et al., 2024). In this study, transcriptome sequencing and non-targeted metabolomics were used to analyze the regulatory mechanism of the difference in the quality of peanut grains during the drying process at five time points of drying by RNA sequencing (RNA-seq) and liquid chromatography-tandem mass spectrometry (LC-MS), which provides a theoretical basis to improve the quality of peanut grain.

## 2 Materials and methods

### 2.1 Plant materials and sample collection

**Figure 1:**
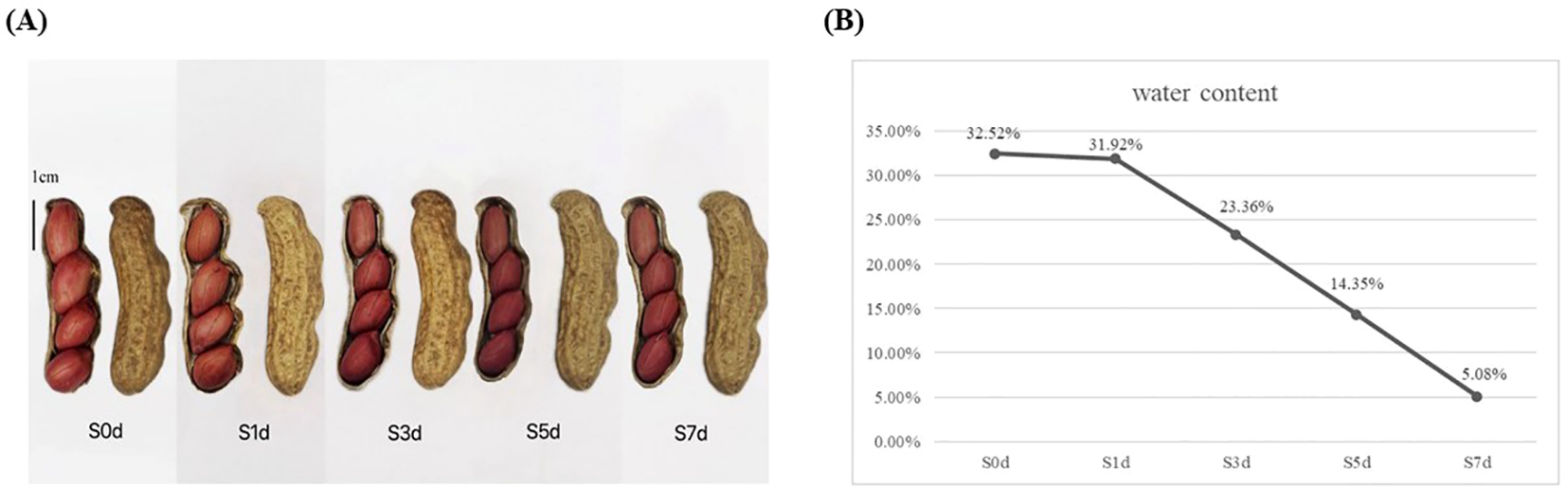
Moisture content and changes in appearance during the drying process of peanut seeds. **(A)** Silihong seeds during the drying process of peanuts. **(B)** Change in the moisture content during the drying process of peanut seeds. The X-axis represents dry days. The Y-axis represents water content.

The peanut variety “Silihong” was obtained from the Peanut Breeding Innovation Team of Hebei Agricultural University (Baoding, China). Silihong was planted in the Xushui Experimental Base (E 1156 13’, N 39 06’) of Hebei Agricultural University in Baoding City, Hebei Province, China, in May 2023. It was harvested in September 2023 and dried at room temperature (temperature 20 °C ~26 °C, humidity 40%~60%, air flow: 0.3m/s) for 0, 1, 3, 5, and 7 days. Ten mature, full, and undamaged seeds were sampled at 0, 1, 3, 5, and 7 days with three replicates for each stage. They were frozen in liquid nitrogen and stored at -80 °C for future transcriptome and metabolomics analyses. The grouping of peanut kernels with different drying days is shown in **Figure 1A**.

### 2.2 Measurement of the moisture content during the drying process of peanut seeds

A total of 50 mature, plump, and undamaged seeds from the samples of five drying stages (S0d, S1d, S3d, S5d, and S7d) were selected and dried in a 60 °C oven for 48 h. The seeds were weighed after drying and cooled to room temperature. Subsequently, the seeds were dried for an additional 24 h and weighed again. If the weight difference between the two drying measurements ≤ 0.002 g, the average of these two weights was recorded as the constant weight of the seed. The percentage of moisture in the seeds was determined by calculating the ratio of the weight of water lost during the drying process to the initial weight of the seeds. The moisture content of the seeds was calculated using the following equation:


$$\text{Seed moisture content }(\%)=\frac{\text{Weight before Drying}-\text{Weight after Drying}}{\text{Weight before Drying}}\times 100\%$$

### 2.3 Analysis of the RNA sequencing data

Total RNA from the peanut kernel samples was extracted using the Tiangen polysaccharide polyphenol plant total RNA extraction kit (Cat. No. DP441; Tiangen Biochemical Technology, Beijing, Co., Ltd., Beijing, China). Stringent quality control measures were implemented to assess the library construction to ensure the success of sequencing. In particular, the RNA was subjected to 1% agarose gel electrophoresis to evaluate its integrity and detect contamination with DNA in the samples. Additionally, a NanoPhotometer spectrophotometer was utilized to measure the purity and concentration of the RNA, while a Qubit 2.0 Fluorometer (Therma Fisher Scientific, Waltham, MA, USA) was employed to quantify the concentration of RNA. Transcriptome sequencing was performed on the Illumina NovaSeq 6000 platform. Its integrity was assessed using an Agilent 2100 Bioanalyzer (Agilent Technologies, Santa Clara, CA, USA). The low-quality data were filtered using fastp v0.23.4 to ensure clean reads for the subsequent analysis (Chen S. et al., 2018). The short read alignment tool Bowtie v2.4.4 was employed to align the clean reads (Langmead and Salzberg, 2012). The ribosome database of the species was used to exclude the ribosome reads that did not permit mismatches. The remaining unmapped reads were then used for an additional transcriptomic analysis. The clean reads were subsequently mapped to the reference genome(NCBI_GCF_003086295.2) using HISAT v2.1.0 software (Kim et al., 2015). The transcripts were reconstructed using StringTie v2.1.7 based on the HISAT v2.1.0 alignment results, and the levels of expression of all the genes in each sample were calculated using RSEM v1.3.1 (Pertea et al., 2015; Li and Dewey, 2011). Differential genes between the groups were enriched using a KEGG (www.kegg.jp/kegg/kegg1.html) analysis conducted with DESeq2 v1.22.1 software. In order to reduce the possibility of increasing the false positive rate, we defined the genes with FDR<0.05 and | log2fc |>log2 (2) as DEGs (Dudoit et al., 2002; Love et al., 2014),. The differential genes were mapped to each term in the Gene Ontology (GO) database (http://www.geneontology.org/), and the number of differential genes associated with each term was calculated to assess the representation of differential genes within specific GO functions. Subsequently, a hypergeometric test was employed to identify the GO terms that were significantly enriched among the differential genes in comparison to the background. All the graphs used in this study were generated using the Omicsmart platform.

### 2.4 Metabolite extraction and quantitation

The samples (100 μL) were placed in Eppendorf tubes and resuspended with prechilled 80% methanol and 0.1% formic acid by vertexing. The samples were then incubated on ice for 5 min and centrifuged at 15,000 g, 4 °C for 20 min. Some of the supernatant was diluted to a final concentration that contained 53% methanol using LC-MS grade water. The samples were subsequently transferred to a fresh Eppendorf tube and then centrifuged at 15,000 g, 4 °C for 20 min. Finally, the supernatant was injected into the LC-MS/MS system (Want et al., 2013). LC-MS/MS analyses were performed using an ExionLC™ AD system (SCIEX) coupled with a QTRAP^®^ 6500+ mass spectrometer (SCIEX) in Genedenovo (Guangzhou, China). Samples were injected onto a Xselect HSS T3 (2.1×150 mm, 2.5 μm) using a 20-min linear gradient at a flow rate of 0.4 mL/min for the positive/negative polarity mode. The eluents were eluent A (0.1% Formic acid-water) and eluent B (0.1% Formic acid-acetonitrile) (Luo et al., 2015). The solvent gradient was set as follows: 2% B, 2 min; 2-100% B, 15.0 min; 100% B, 17.0 min;100-2% B, 17.1 min;2% B, 20min. QTRAP^®^ 6500+ mass spectrometer was operated in positive polarity mode with Curtain Gas of 35 psi, Collision Gas of Medium, Ion Spray Voltage of 5500V, Temperature of 550°C, Ion Source Gas of 1:60, Ion Source Gas of 2:60. QTRAP^®^ 6500+ mass spectrometer was operated in negative polarity mode with Curtain Gas of 35 psi, Collision Gas of Medium, Ion Spray Voltage of -4500V, Temperature of 550°C, Ion Source Gas of 1:60, Ion Source Gas of 2:60. The detection of the experimental samples using Multiple Reaction Monitoring (MRM) was based on the house database. The Q3 was used to quantify the metabolites. The Q1, Q3, RT (retention time), DP (declustering potential) and CE (collision energy) were used to identify the metabolites. The data files generated by LC-MS/MS were processed using SCIEX OS Version 1.4 (SCIEX, Framingham, MA, USA) to integrate and correct the peak. The main parameters were set as follows: minimum peak height, 500; signal/noise ratio, 5; and Gaussian smooth width, 1. The area of each peak represents the relative content of the corresponding substance.

### 2.5 Metabolomics data analysis

PCA analysis is an unsupervised, multidimensional statistical analysis method that reflects the overall metabolic differences between groups and the magnitude of variability between samples within groups. The analysis was performed using R package gmodels (Warnes et al., 2015). The parameters R²X, R²Y, and Q² of the PLS-DA model were employed to evaluate the reliability of the model (Worley and Powers, 2013). To further validate the reliability of the OPLS-DA model, cross-validation and permutation tests were performed. Differential metabolites (DAMs) among the various comparison groups were identified by integrating the variable importance in projection (VIP) values derived from the multivariate statistical OPLS-DA with the p-values obtained from a univariate statistical t-test (Bylesjö et al., 2006). Finally, in order to screen out dams that have important contributions to model discrimination, but also have significant differences between groups and are more reliable, we combined OPLS-DA (VIP ≥ 1, t test p<0.05) to screen dams between different control groups (Xia et al., 2009; Saccenti et al., 2014).

### 2.6 Weighted gene coexpression network analysis

Using the Omicsmart (https://www.omicsmart.com/#/) platform for weighted gene co-expression network (WGCNA) analysis, low-expression genes (count < 10) were filtered out to avoid including spurious edges in the network. Set soft thresholding power=7 module merging criteria=0.15 to divide modules. Based on this strategy, the remaining 19,078 genes were used for correlation analysis with DAMs (amino acids and their derivatives (51), followed by lipids (25), carbohydrates and their derivatives (21), and flavonoids (15)) to identify modules with high correlation coefficients with DAMs and screen for key genes.

### 2.7 Combined analysis of the transcriptome and metabolome

A Pearson analysis was conducted on the selected DEGs and DAMs during the process of seed kernel drying. The DEGs with an absolute correlation coefficient > 0.8 and P<0.05 were selected for further analysis. Additionally, a KEGG pathway analysis was performed, and a pathway diagram was created.

### 2.8 Real-time fluorescence quantitative PCR validation

Nine DEGs were validated using real-time quantitative reverse transcription PCR (qRT-PCR). Specific primers for the DEGs were designed using Primer Premier 5.0 software (Primer, Canada). Alcohol dehydrogenase (ADH) was used as the internal reference gene, and the relative levels of expression of the DEGs were measured. The primer sequences utilized for qRT-PCR are provided in **Supplementary Table 1**. The qRT-PCR reactions were conducted using a Fast Real qPCR Premix (SYBR Green) kit (TIANGEN, Beijing, China). Each RT-qPCR reaction system was composed of a volume of 20 µL, including 0.6 µL of upstream and downstream primers, 4 µL of cDNA template, 10 µL of SYBR reagent, and 4.8 µL of ddH2O. The PCR program included an initial pre-denaturation step at 95 °C for 10 min, followed by thermal cyclings: denaturation at 95 °C for 5 s, annealing at 58 °C for 10 s, and an extension at 72 °C for 15 s, which was repeated for a total of 40 cycles. A LightCycler^®^ 96 SW 1.1 (Roche Diagnostics, Rotkreuz, Switzerland) was used to read the qPCR results, and the relative levels of expression of the genes were calculated using the 2_−ΔΔCT_ method.

## 3 Results

### 3.1 Moisture content during the drying process of the peanut seeds

During the drying process of the mature peanut kernels, the moisture content of the seeds gradually decreased as the number of drying days increased. On the first day of drying, the initial moisture content of the peanut seeds was notably high and reached 32.52%. However, after 1 day of drying, the moisture content was minimally reduced and decreased to 31.92%. As the number of days of drying increased, the moisture content began to decrease rapidly. By the third day of drying, the moisture content had decreased significantly to 23.36%. When the fifth day of the drying process arrived, the moisture content had decreased further to 14.35%. Ultimately, on the seventh day of drying, the moisture content stabilized at 5.08% (**Figure 1B**). The moisture content in the peanut seeds must be < 8% to meet the storage standards (de Oliveira Sá et al., 2020).

### 3.2 Transcriptome analysis during the drying process of the peanut seeds

A total of 98.01 Gb of raw reads were obtained through the transcriptome sequencing analysis of the peanut kernels during the drying process, which yielded 84.86 Gb of clean reads after the removal of the reads that contained the connectors and those deemed low quality. The clean reads for each sample exceeded 5.96 Gb, with Q20 and Q30 values that surpassed 98.79% and 96.44%, respectively. The clean sequencing reads were aligned with the reference genome after the raw data had been filtered and used to analyze the distribution of the GC content. More than 81.78% of the reads were uniquely mapped to the reference genome, and more than 91.53% of the reads were found within the exon regions. These reference data indicate that the sequencing results were highly accurate and suitable for further analysis (**Table 1**).

**Table 1:** Statistical analysis of the RNA-seq data quality.

Sample	Raw Data (bp)	Clean Data (bp)	AF_Q20 (%)	AF_Q30 (%)	AF_GC (%)	Unique_Mapped(%)	exon(%)
S0d-1	7443542400	7358538148	98.96	96.88	45.34	82.00	90.45
S0d-2	6958143600	6859521247	98.95	96.87	45.08	81.78	89.90
S0d-3	8223071400	8121768014	98.99	96.99	45.43	81.87	90.93
S1d-1	7344545400	7247418210	98.96	96.91	45.63	83.31	92.57
S1d-2	6779460300	6703653471	99.02	97.07	45.40	83.31	91.46
S1d-3	7648719900	7556278799	99.11	97.35	45.57	82.69	91.93
S3d-1	6256423200	6187543591	98.86	96.61	45.13	83.13	91.50
S3d-2	6816291600	6733989224	98.80	96.45	45.03	83.39	92.01
S3d-3	7566775800	7473194283	98.88	96.71	45.16	83.22	92.02
S5d-1	6044081100	5965090633	98.81	96.49	45.03	83.16	91.71
S5d-2	7107005400	7015895334	98.82	96.50	45.29	82.48	92.18
S5d-3	6193295400	6128765298	98.86	96.63	45.30	82.87	92.33
S7d-1	7660830300	7547232088	99.05	97.15	45.04	82.18	91.20
S7d-2	7039206900	6946917273	98.79	96.57	45.13	82.71	92.05
S7d-3	6037562700	5957331907	99.00	97.00	44.99	83.06	90.69

**Figure 2:**
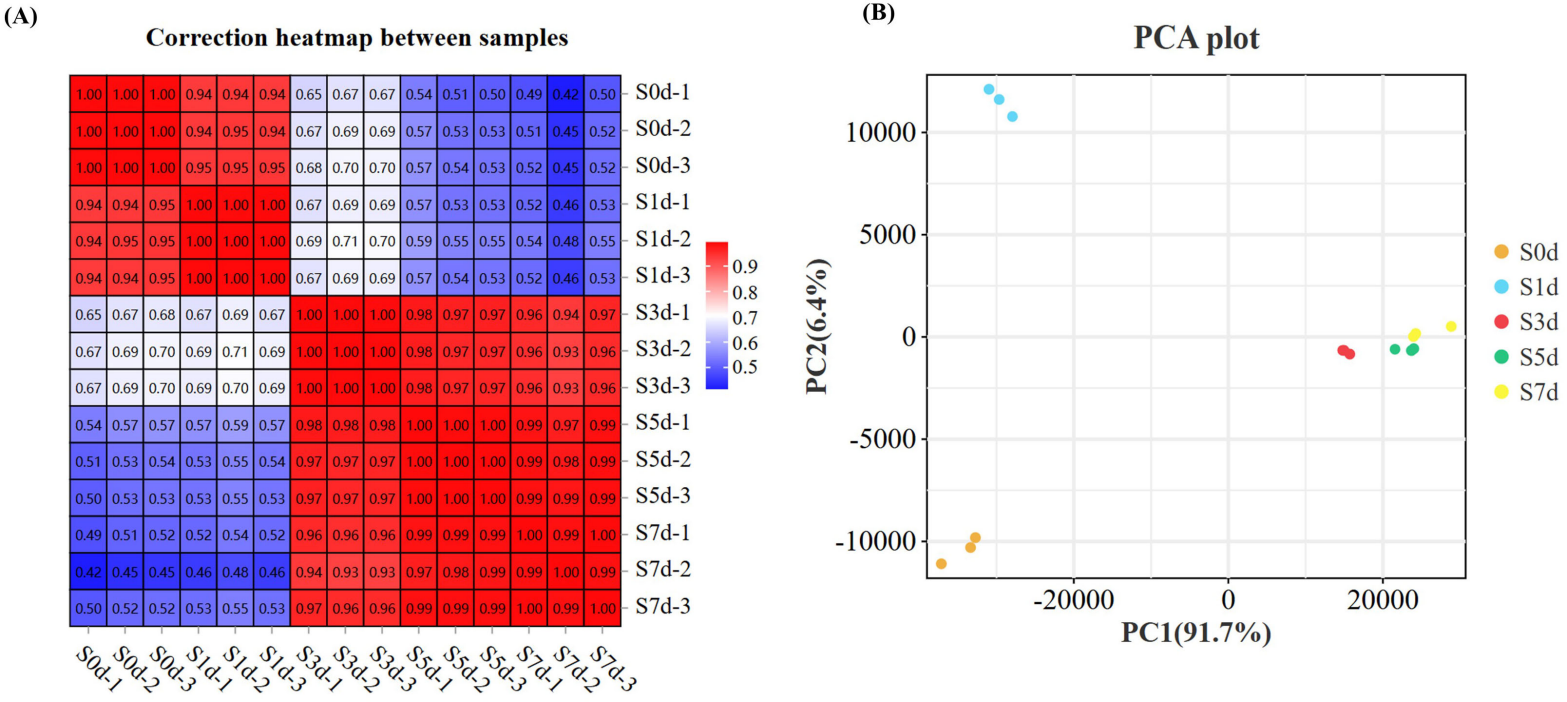
Transcriptome analysis during the drying process of peanut seeds. **(A)** Correlation heatmap between the samples. **(B)** PCA analysis between the samples. PCA, Principal component analysis.

The correlation among the three replicates of all test samples > 0.99 (**Figure 2A**). Principal Component 1 (PC1) and Principal Component 2 (PC2) accounted for 91.7% and 6.4% of the variation, respectively (**Figure 2B**), which indicated a significant change in the levels of the transcriptome of the peanut kernels during the drying process.

### 3.3 Analysis of the differential genes during the drying process of peanut seeds

**Figure 3:**
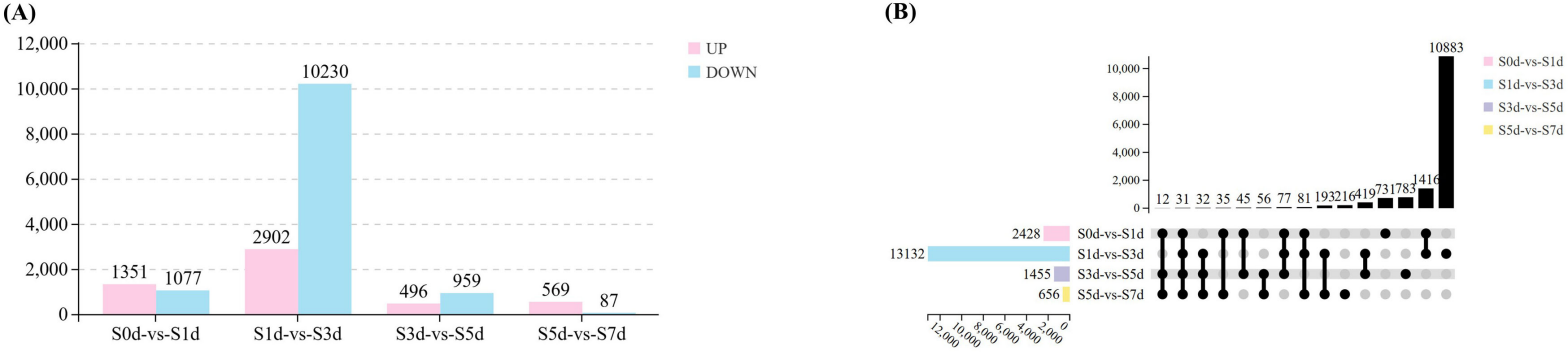
DEGs during the drying process of peanut seeds. **(A)** Statistical charts of four comparative groups of DEGs. **(B)** Upset diagram of the DEGs in four comparative groups.

To verify the dynamic changes during the drying process, four comparison groups (S0d-vs-S1d; S1d-vs-S3d; S3d-vs-S5d; S5d-vs-S7d) were selected for subsequent analysis. During the seed drying process, a total of 15,010 DEGs exhibited changes in expression. The most significant alterations in gene expression occurred in various metabolic pathways at the S3d. In comparison to S0d, we identified 2,428 DEGs at S1d, with 1,351 upregulated and 1,077 downregulated. When S1d was compared to S3d, we identified 13,132 DEGs, including 2,902 that were upregulated and 10,230 that were downregulated. In the comparison from S3d to S5d, 1,455 DEGs were identified, with 496 upregulated and 959 downregulated. Finally, a comparison of S5d with S7d revealed 656 DEGs, of which 569 were upregulated and 87 were downregulated (**Figure 3A**; **Supplementary Figure 1**, **Supplementary Table 2**). The UpSet diagram illustrates that 731, 10,883, 783, and 216 DEGs were differentially expressed in the S0d-vs-S1d, S1d-vs-S3d, S3d-vs-S5d, and S5d-vs-S7d comparison groups, respectively. Additionally, 31 DEGs were consistently expressed across all four comparison groups (**Figure 3B**).

**Figure 4:**
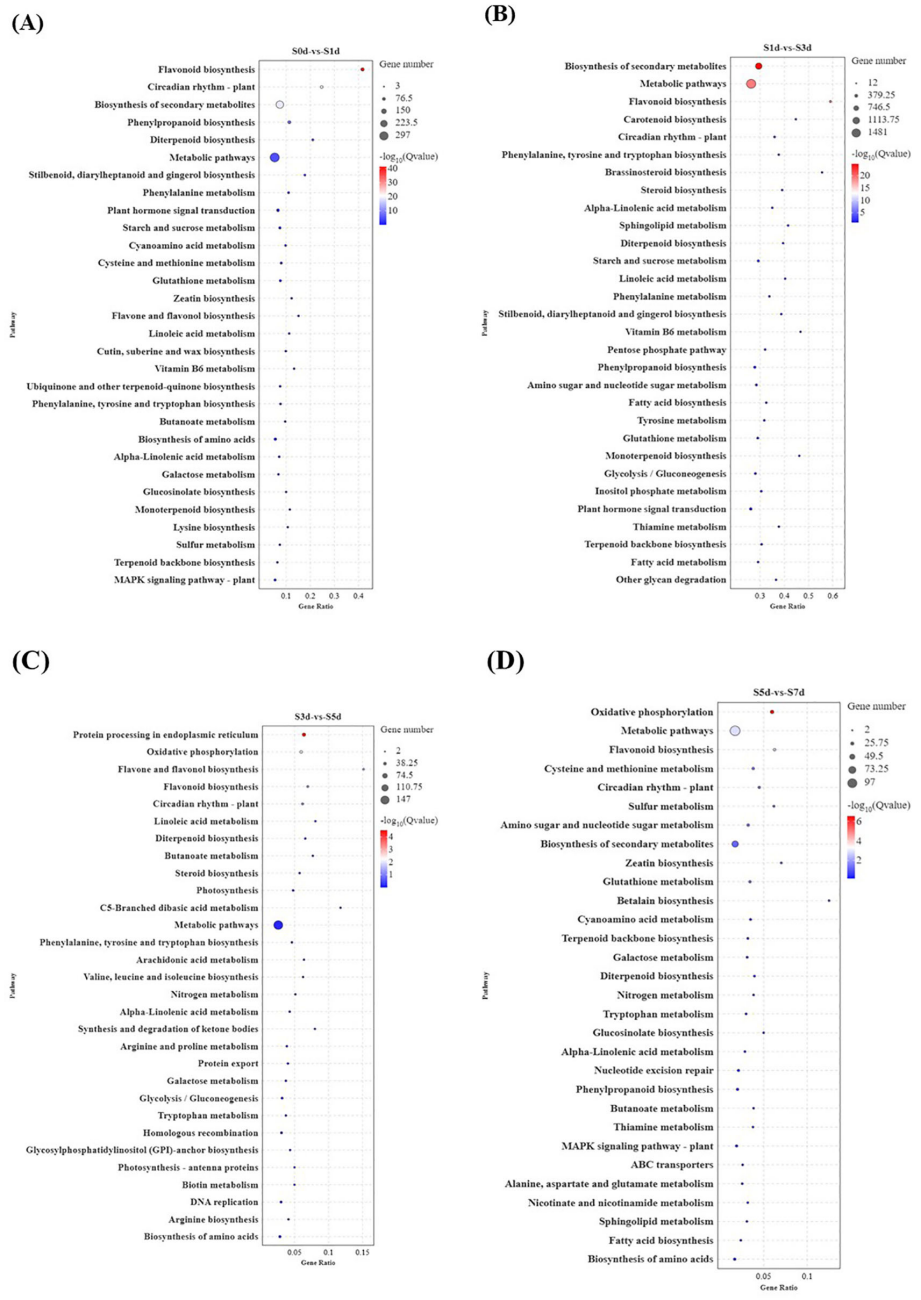
KEGG pathway analysis of DEGs during the drying process of peanut seeds. **(A)** KEGG enrichment analysis of S0d-vs-S1d. **(B)** KEGG enrichment analysis of S1d-vs-S3d. **(C)** KEGG enrichment analysis of S3d-vs-S5d. **(D)** KEGG enrichment analysis of S5d-vs-S7d. DEGs, differentially expressed genes; KEGG, Kyoto Encyclopedia of Genes and Genomes.

To further elucidate the properties and biological functions of the genes and their products in organisms, GO and KEGG pathway enrichment analyses were employed for annotation. The GO cluster analysis revealed that the DEGs in the comparative groups were predominantly categorized into three main areas, including biological processes (BPs), molecular functions (MFs), and cellular components (CCs) (**Supplementary Figure 2**). The top 20 KEGG pathways were plotted based on their lowest p-values with a significance threshold of p < 0.05. The analysis revealed 19, 46, 8, and 13 significant pathways across the four comparison groups, respectively. In the four comparative analysis groups, the KEGG pathways were primarily enriched in four key areas, including amino acid metabolism, lipid metabolism, starch and sucrose metabolism, and flavonoid biosynthesis (**Figures 4A–D**). Notably, the biosynthesis of flavonoids was significantly enriched across all four groups. It is notable that the S1d-vs-S3d comparison was notable as the stage with the highest number of enriched entries, the largest count of DEGs, and the most vigorous metabolic activity. Furthermore, it marked a critical period during which substantial changes occurred in the storage substances, including amino acids, lipids, and sugars. The KEGG pathway and GO analyses showed that the DEGs primarily influenced the quality of peanuts by affecting the levels of sugars, amino acids, lipids, and flavonoids as the kernels dried.

### 3.4 Metabolome analysis during the drying process of the peanut seeds

**Figure 5:**
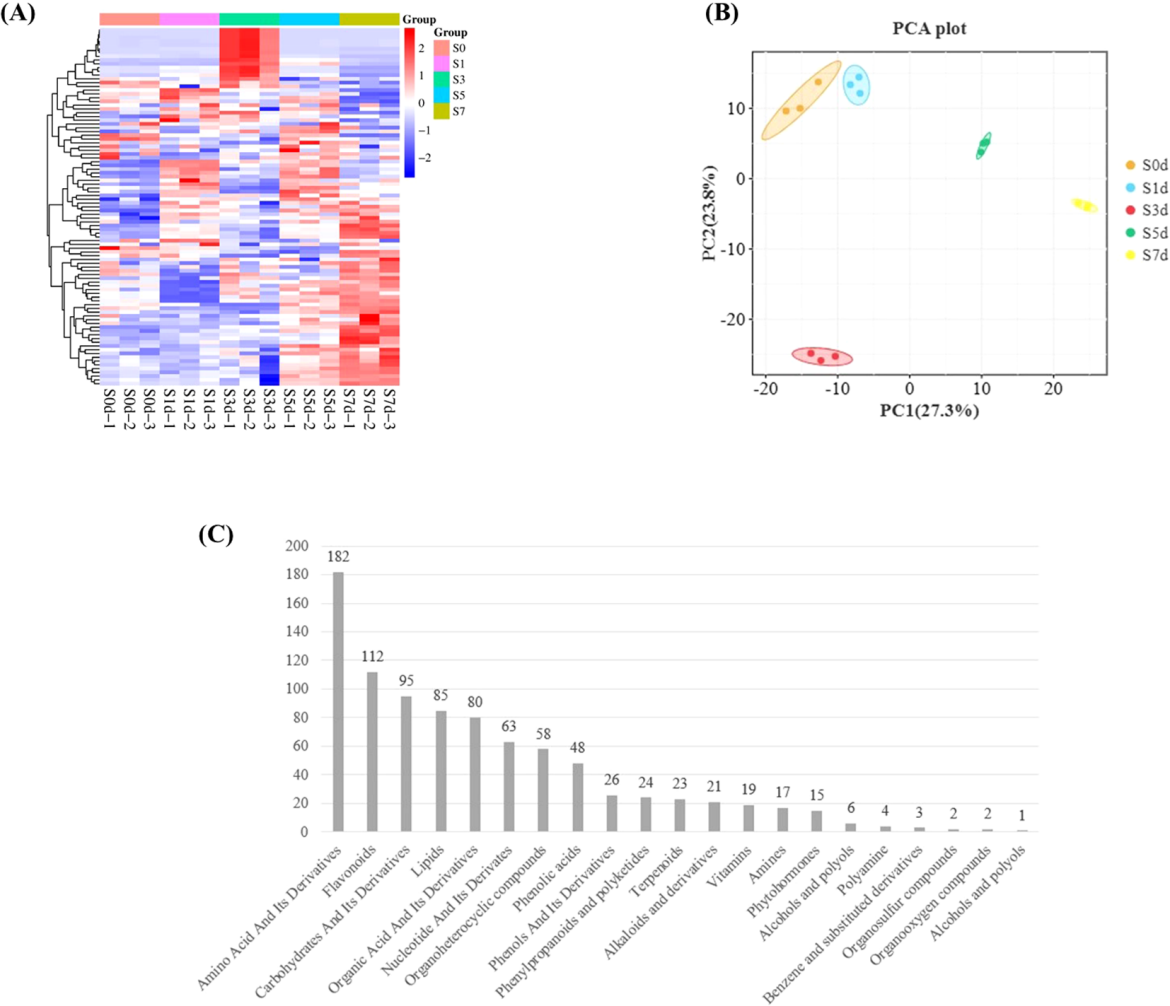
Metabolome analysis during the drying process of peanut seeds. **(A)** Metabolite clustering heatmap. The X-axis represents the kind of metabolite. The Y-axis represents the number of metabolites. **(B)** Sample PCA analysis chart. **(C)** Metabolite statistics. PCA, Principal component analysis.

To investigate the metabolic network during the drying process of peanut seeds, we employed LC-MS-based metabolomics to conduct a metabolic analysis at five distinct time points throughout this process. The PLS-DA model was utilized to analyze and score each comparison group, with all Q2 values > 0.99, which indicated that the model was highly credible and suitable. Additionally, the Q2 and Q2Y values of the OPLS-DA model were both > 0.9 in each comparison group (**Supplementary Table 3**). These results demonstrated that both the PLS-DA and OPLS-DA models are highly reliable and appropriate for evaluating differences among the four groups. Cluster analysis heat map indicates that the sample groups were significant differences in the metabolites between the groups (**Figure 5A**). The PCA plot revealed that the first principal component accounted for 27.3% of the variance, while the second principal component accounted for 23.8%, which resulted in a cumulative variance explanation of 51.1% for the dataset (**Figure 5B**). The metabolome analysis identified a total of 886 metabolites, which were categorized into 21 groups. Among these, there were 182 amino acids and their derivatives, followed by 112 flavonoids, 95 carbohydrates and their derivatives, 85 lipids, and 80 organic acids and their derivatives (**Figure 5C**; **Supplementary Table 4**).

### 3.5 Analysis of the differential metabolites during the drying process of the peanut seeds

**Figure 6:**
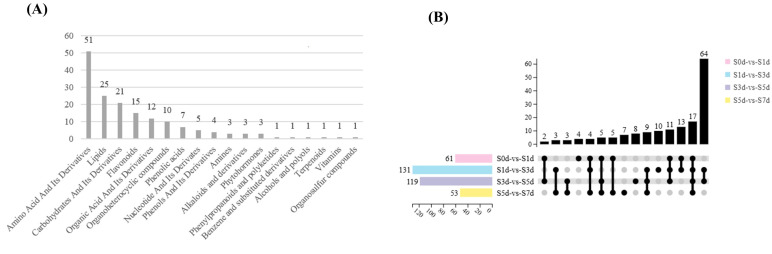
**(A)** Statistical charts of the differential metabolites in the four comparison groups. The X-axis represents the kind of metabolite. The Y-axis represents the number of metabolites. **(B)** Upset diagram of the differential metabolites in the four comparison groups. The X-axis represents the four comparison groups. The Y-axis represents the number of metabolites.

A total of 165 metabolites were detected and classified into 21 categories. The compounds that were represented the most included amino acids and their derivatives (51), followed by lipids (25), carbohydrates and their derivatives (21), and flavonoids (15) (**Figure 6A**). In the four comparison groups, the number of differential metabolites showed a trend of increasing first and then decreasing. In particular, S1d-vs-S3d had the highest number of DAMs, with a total of 131 DAMs screened, including 78 upregulated metabolites and 53 downregulated metabolites. In contrast, the fewest DAMs were found in S5d-vs-S7d with only 53, of which 24 were upregulated and 29 downregulated (**Figure 6B**; **Supplementary Table 5**).

**Figure 7:**
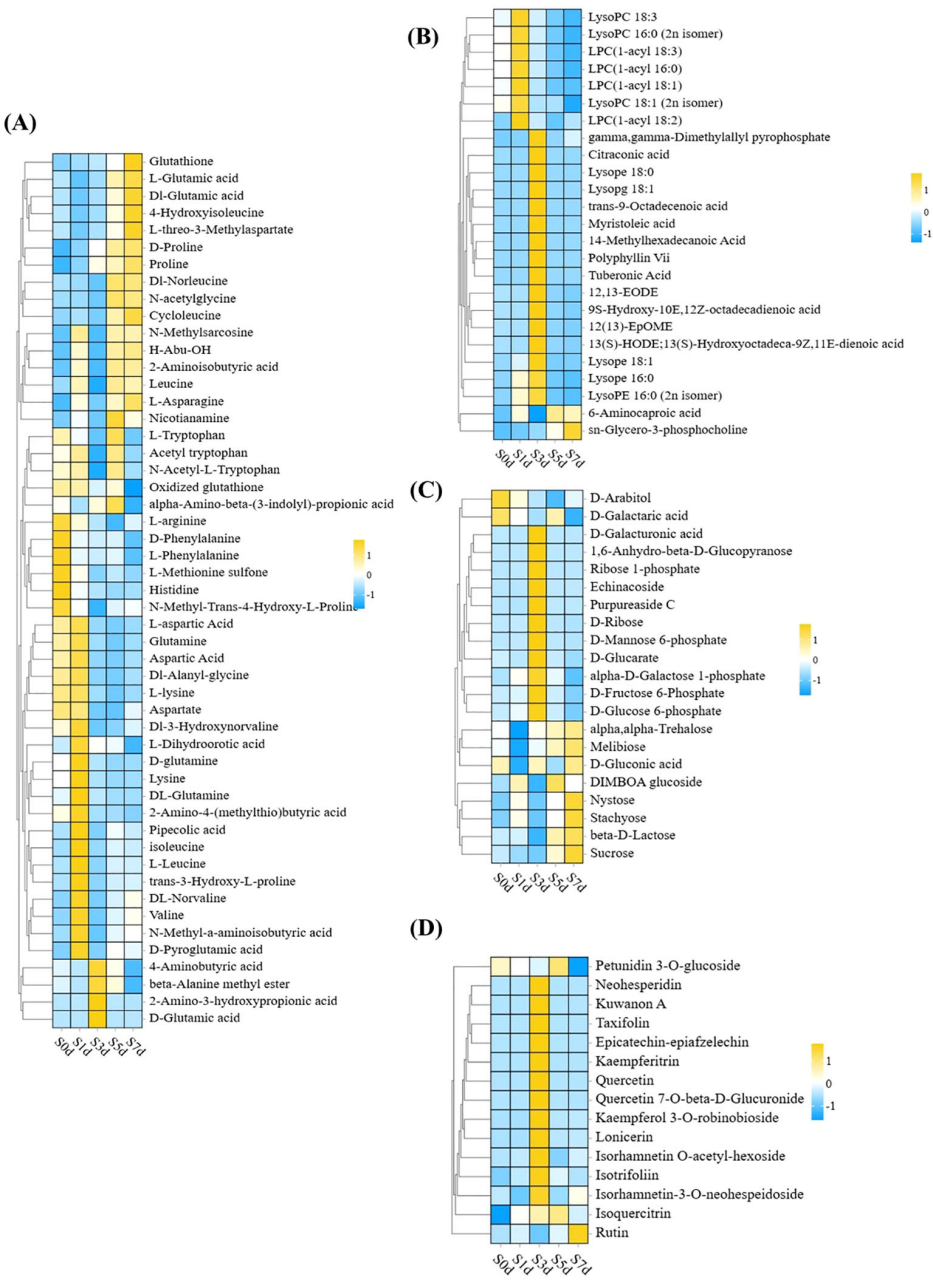
Cluster heat map of four classes of DAMs: amino acids and their derivatives, lipids, carbohydrates and their derivatives, and flavonoids **(A)** Heatmap of the differential metabolites for amino acids and their derivatives. **(B)** Heatmap of the lipid differential metabolites. **(C)** Heatmap of the differential metabolites of carbohydrates and their derivatives. **(D)** Heatmap of the differential metabolites of flavonoids. KEGG, Kyoto Encyclopedia of Genes and Genomes. The X-axis represents five peanut samples from different periods, and the Y-axis represents DAMs.

In this study, 51 types of amino acids were primarily characterized as glycogenic and ketogenic amino acids that predominantly accumulated during various periods with the exception of S3d. In particular, glycogenic amino acids, such as L-arginine, D-glutamic acid and L-aspartic acid, as well as ketone amino acids, including isoleucine, L-leucine, and L-lysine, accumulated significantly during the early stages of drying (S0d and S1d). A small portion of glycogenic amino acids, namely L-glutamic acid and proline, accumulated at S7d (**Figure 7A**; **Supplementary Table 5**).

Among the 25 lipids that were meticulously selected, the preponderance accumulated the most at S1d and S3d and was primarily composed of lysophospholipids and long-chain fatty acids. Furthermore, 6-aminocaproic acid and sn-glycero-3-phosphocholine attained their maximal levels of accumulation at S5d and S7d, respectively (**Figure 7B**; **Supplementary Table 5**).

Carbohydrates and their derivatives are also primary metabolites that undergo significant changes, with S3d and S7d emerging as particularly pivotal stages for the accumulation of sugar. In particular, during the S3d phase, the sugar intermediates, including D-glucose-6-phosphate, D-fructose-6-phosphate, and D-mannose-6-phosphate, accumulated to considerable levels. Trehalose, β-D-lactose, and sucrose accumulated markedly at S7d (**Figure 7C**; **Supplementary Table 5**).

In addition to the primary metabolites, the secondary metabolites also changed significantly. Among them, the flavonoids predominated, with a total of 15 species identified. Notably, 12 of these species accumulated significantly at S3d. They primarily consisted of flavonol metabolites with quercetin as the core structure. Additionally, only rutin reached its maximum level of accumulation at S7d (**Figure 7D**; **Supplementary Table 5**).

### 3.6 WGCNA

**Figure 8:**
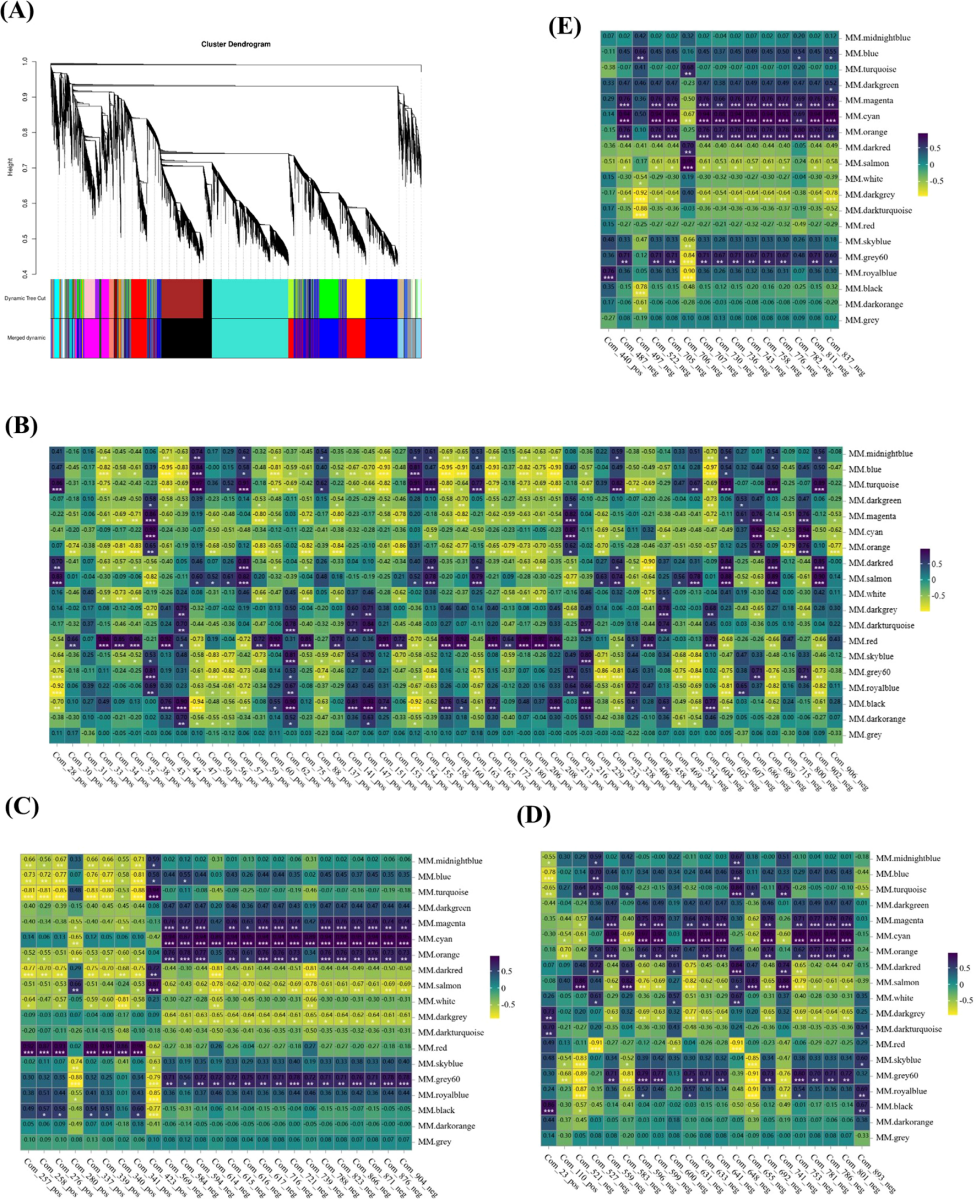
Weighted gene co-expression network analysis (WGCNA) of genes during the drying process of peanut seeds. **(A)** Modules level clustering diagram. **(B)** Heatmap of correlation between 51 amino acids and their derivatives and modules. **(C)** heatmap of correlation between 25 lipids and modules. **(D)** Heatmap of correlation between 21 Carbohydrates and their derivatives and modules. **(E)** Heatmap of correlation between 15 flavonoids and modules. * At the 0.05 level, the correlation is significant; **At the 0.01 level, the correlation is significant; *** At the 0.001 level, the correlation is significant.

Through the construction of a gene co-expression network, we identified gene modules and pivotal genes, with the objective of elucidating the intergenic correlations and shared regulatory mechanisms. Employing the WGCNA methodology, a total of 19,078 genes were meticulously partitioned into 19 distinct modules (**Figure 8A**). Subsequent correlation analysis between these modules and 165 DAMs (consisting of 51 amino acids and their derivatives, 25 lipids, 21 carbohydrates and their derivatives, as well as 15 flavonoids) indicated that correlations with absolute coefficient values exceeding 0.8 were predominantly observed in the red and cyan modules (**Figures 8B–E**). Specifically, the red module exhibited strong correlations with 51 amino acids and their derivatives, whereas the cyan module showed robust correlations with 25 lipids, 21 carbohydrates and their derivatives, and 15 flavonoids. Within the red and cyan modules, 2,970 and 326 genes were identified, respectively (**Supplementary Table 6**). Notably, the majority of genes within the red module were highly expressed during the S1d stage, with KEGG pathway analysis revealing significant enrichment in oxidative phosphorylation, ribosome, and amino acid biosynthesis. In contrast, the majority of genes within the cyan module were highly expressed during the S3d stage, with KEGG pathway analysis highlighting enrichment in ribosome, galactose metabolism, and alanine, aspartate, and glutamate metabolism (**Supplementary Figures 3**, **4**).

### 3.7 Combined analysis of the transcriptome and metabolome

To further investigate the regulatory mechanism of the changes in quality during the drying process of peanut seeds, we conducted a correlation analysis between the DEGs and DAMs. A total of 10,078 DEGs, which had correlation coefficients > 0.8 and p-values < 0.05 with 165 DAMs, were screened out and subjected to a KEGG enrichment analysis. This analysis aimed to explore the potential associations between the metabolome and transcriptome during the drying process of peanut seeds. The KEGG pathway analysis revealed that these 10,078 DEGs were primarily enriched in four aspects, including amino acid metabolism, lipid biosynthesis, sucrose and starch metabolism, as well as flavonoid biosynthesis (**Supplementary Figure 5** and **Supplementary Table 7**).

**Figure 9:**
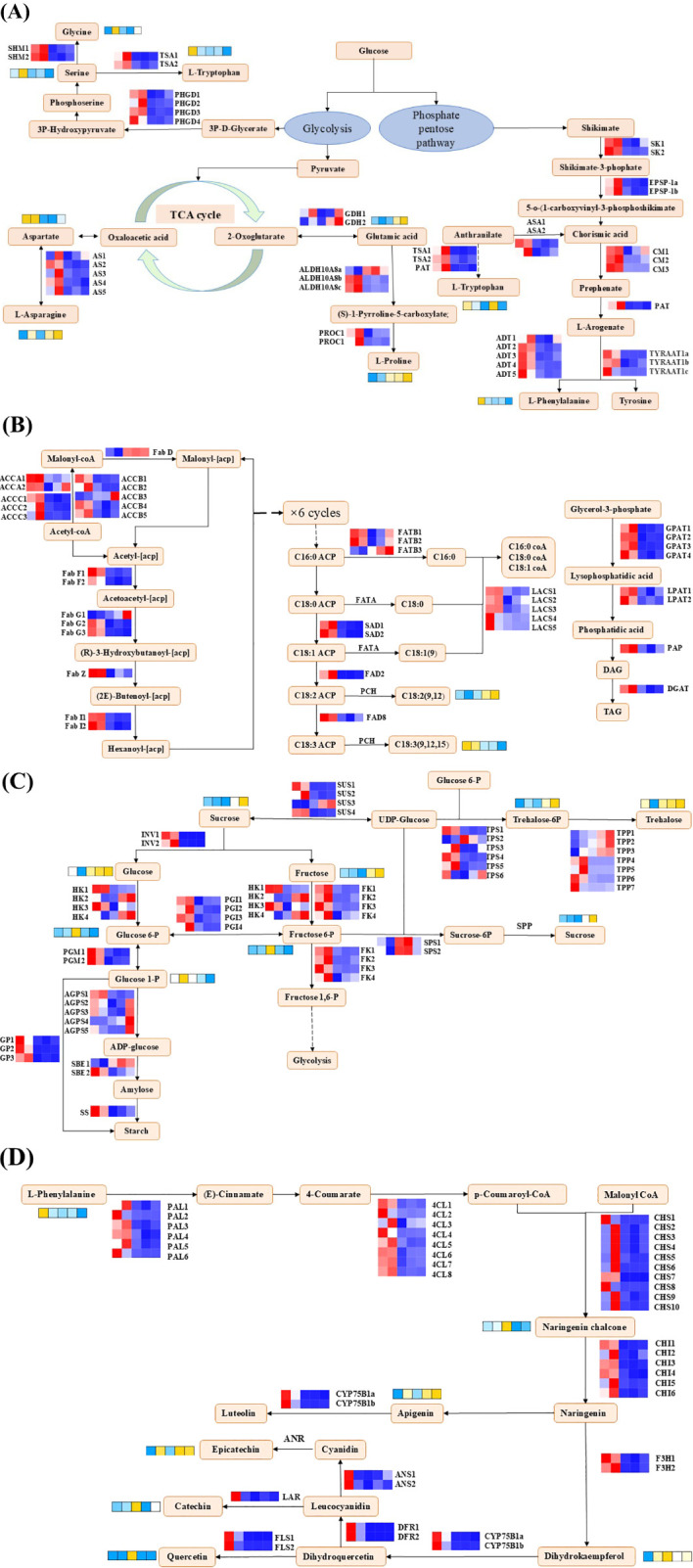
The profiles of expression of the genes and metabolites. **(A)** Amino acid biosynthesis and metabolism. **(B)** Lipid biosynthesis. **(C)** Starch and sucrose metabolism. **(D)** Flavonoid biosynthesis. *SK*, shikimate kinase; *EPSP-1*, 3-phosphoshikimate 1-carboxyvinyltransferase; *ASA*, anthranilate synthase component I; *CM*, chorismate mutase; *PAT*, bifunctional aspartate aminotransferase; *ADT*, arogenate/prephenate dehydratase; *TSA*, tryptophan synthase; *TYRAAT1*, arogenate dehydrogenase; *AS*, asparagine synthase; *GDH*, glutamate dehydrogenase; *ALDH18A1*, Δ-1-pyrroline-5-carboxylate synthase; *ProC*, pyrroline-5-carboxylate reductase; *SHM*, serine hydroxymethyltransferase; *PHGD*, D-3-phosphoglycerate dehydrogenase; *ACCA*, acetyl CoA carboxylase; *FabD*, acyl-carrier-protein S-malonyltransferase; *FabF*, 3-oxoacyl-[acyl-carrier-protein] synthase II; *FabG*, 3-oxoacyl-[acyl-carrier-protein] reductase; *FabZ*, 3-hydroxyacyl-[acyl-carrier-protein] dehydratase; *FabI*, enoyl-[acyl-carrier-protein] reductase; *FATB*, fatty acyl carrier protein thioesterase B; *SAD*, stearoyl-acyl carrier protein Δ^9^ desaturases; Oleate dehydrogenase, *FAD2*; fatty acid desaturase 8, *FAD8*; *LACS*, long-chain acyl CoA synthetase; *GPAT*, glycerol-3-phosphate acyltransferase; *LPAT*, lysophosphatidic acid acyltransferase; *DGAT*, diacylglycerol acyltransferase; fatty acyl-ACP thioesterase A, *FATA*; *SUS*, sucrose synthase; *INV*, invertase; *HK*, hexokinase; f *FK*, ructokinase; PGI, glucose-6-phosphate isomerase; *PGM*, phosphoglucomutase; *AGPS*, glucose-1-phosphate adenylyltransferase; *SBE*, starch branching enzyme; *SS*, starch synthase; *GP*, glycogen phosphorylase; *TPS*, trehalose-6-phosphate synthase; *TPP*, trehalose-6-phosphate phosphatase; *PAL*, phenylalanine ammonia-lyase; *4CL*, 4-coumaroyl-CoA ligase; *CHS*, chalcone synthase; *CHI*, chalcone isomerase; *F3H*, naringenin 3-dioxygenase; *CYP75B2*, flavonoid 3’-monooxygenase; *DFR*, dihydroflavonol 4-reductase; *ANS*, anthocyanidin synthase; *LAR*, leucoanthocyanidin reductase.

The primary components in the amino acid metabolic pathway were the intermediates of the tricarboxylic acid (TCA) cycle, the shikimic acid pathway, and glycolysis-derived amino acids from triose-3-phosphate. Various amino acids accumulated significantly during S0d and S1d. Proline and glutamic acid exhibited a pattern of inverse accumulation compared to the others. In the early stages of drying (S0d and S1d), related genes (ASs, GDHs, ALDH18A1s, Pro Cs, TSAs, PAT, ADTs, TYRAATs, CMs, EPSP-1s, SKs, SHMs, and PHGDs) involved in the biosynthesis of amino acids were highly expressed. It is worth noting that one ALDH18A1 (loc112722399) and two GDHs (loc112696998, loc112716863) were highly expressed in the glutamate biosynthetic pathway at the late stage of drying (S5d and S7d). This result may be attributed to the functional differences among the different members within the gene family, and the patterns of expression of these three genes were consistent with the patterns of accumulation of glutamic acid and proline (**Figure 9A**; **Supplementary Table 8**).

The genes involved in lipid biosynthesis in this study, such as acetyl CoA carboxylase (ACCAs, ACCBs, ACCCs), various acyl-carrier-proteins (FabD, FabFs, FabGs, FabZ, FabIs, FATBs, SADs), fatty acid desaturase (FAD2, FAD8), long-chain acyl CoA synthetase (LACSs), various acyltransferase (GPATs, LPATs), and diacylglycerol acyltransferase (DGAT), were highly expressed during the early stage of drying. In contrast, one ACCB, loc112783193 and one FabG (loc112697009) were predominantly expressed in S7d. It is worth noting that the pattern of expression of FAD8 was the same as the pattern of accumulation of α-linolenic acid but opposite to the pattern of accumulation of linoleic acid (**Figure 9B**; **Supplementary Table 8**).

In the starch and sucrose metabolic pathways, glucose, fructose, and sucrose all reached their maximum levels of accumulation at S7d. Concurrently, sugar intermediates, such as glucose-6-phosphate, fructose-6-phosphate, and glucose-1-phosphate, accumulated significantly during the mid-stages of the drying process (S1d and S3d). The patterns of expression of the genes involved in starch and sucrose metabolism differed significantly. GPs, PGIs, SS and FKs were highly expressed during the initial drying, which potentially aided the accumulation of starch. Furthermore, different members within the same gene family exhibited distinct patterns of expression. In this study, we conducted a comprehensive identification and discovered a total of seven TPPs and four SUSs. Among them, four TPPs and three SUSs were expressed during the early drying phase, while the remaining three TPPs genes and one SUS gene were expressed during the later drying phase. Notably, among the genes expressed during the later drying phase, the patterns of expression of the SUS and TPPs genes were consistent with the patterns of accumulation of sucrose and trehalose, which suggested that these genes may play a crucial role in the accumulation of these sugars during the later stages of drying (**Figure 9C**; **Supplementary Table 8**).

For flavonoid biosynthesis, major metabolites, such as naringenin chalcone, apigenin, and epicatechin, were remarkably increased at S3d, in contrast to their precursor phenylalanine. In this study, the expression of almost all the selected DEGs involved in the flavonoid metabolism pathway peaked at S0d and S1d at S0d and S1d. The specific patterns of expression of these genes were the key factors that led to the high accumulation of the differential metabolites of flavonoids, such as epicatechin, catechin, and quercetin, at S3d (**Figure 9D**; **Supplementary Table 8**).

### 3.8 Real-time fluorescence quantitative PCR validation

**Figure 10:**
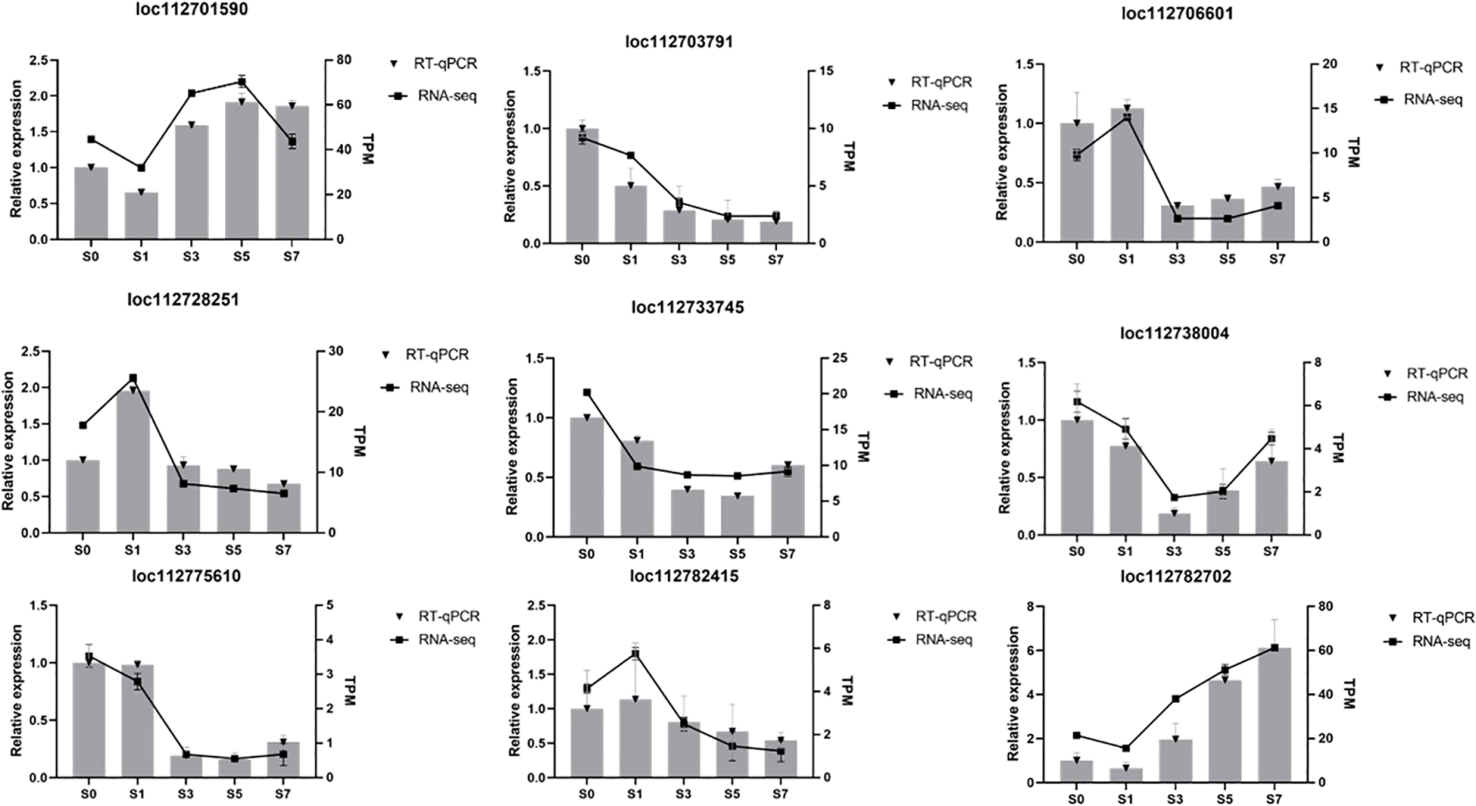
Verification of the level of transcription of the genes using qRT-PCR. qRT-PCR, real-time quantitative reverse transcription PCR. Relative expression is expressed as a fold change relative to S0d. Relative gene expression was calculated using the comparative 2_-ΔΔCT_ method.

To verify the reliability of the transcriptome results and the accuracy of transcriptome data, nine DEGs were subjected to transcriptome sequencing. These genes included loc112701590 (sucrose-phosphate synthase), loc112733745 (receptor protein kinase), loc112738004 (calmodulin-binding protein 60 E), loc112775610 (alpha,alpha-trehalose-phosphate synthase), and loc112782702 (palmitoyl-acyl carrier protein thioesterase), which exhibited an initial decline in expression levels followed by a subsequent increase. Conversely, the expression patterns of loc112706601 (fructokinase), loc112728251 (3-phosphoshikimate 1-carboxyvinyltransferase), and demonstrated an initial surge, followed by a decrement. Notably, the expression level of loc112782415 (anthranilate phosphoribosyltransferase). Notably, the expression level of loc112703791 (3-isopropylmalate dehydrogenase) continued to decline consistently throughout the observed period. The trend of variation of these nine DEGs was consistent with the results obtained from RNA-seq, thereby confirming the accuracy and reliability of the RNA-seq findings (**Figure 10**).

## 4 Discussion

### 4.1 Moisture loss and material transformations during the drying process of the seeds

The developmental stage of seeds is a crucial period for the accumulation of storage compounds, and it is characterized by intricate and complex changes in materials. These changes are primarily evidenced by increases in the contents of proteins and fat along with a decrease in the content of soluble sugar (Wang et al., 2018; Li et al., 2021; Yin et al., 2013). As the seeds enter the drying process post-maturity, moisture loss plays a pivotal role in the transition from the developmental stage to germination and growth (Kermode and Bewley, 1985). During natural drying, the rate of water loss in seeds initially decreases rapidly, followed by a slower phase (Franke et al., 2008). This study showed that beginning at S1d, there was a significant decrease in the moisture content in the seeds (**Figure 1B**), with the period from S1d to S3d representing the most intense stage of internal material changes within the seeds. During this interval, the contents of amino acids and their derivatives decreased, whereas the contents of lipids, sugars and their derivatives, and flavonoids increased (**Supplementary Figure 6**). It is important to note that the drying process of seeds is not merely a loss of water. Rather, it is a complex process that involves the active expression of genes and vigorous metabolism as shown by the transcriptome enrichment analysis (**Figures 4A–D**).

In addition, the changes in materials were particularly pronounced in the comparison between the S1d and S3d groups, with significant enrichment in the pathways, such as amino acid metabolism and biosynthesis fatty acid biosynthesis, starch and sucrose metabolism, and flavonoid biosynthesis, which are all closely related to the changes in metabolites. This suggests that the period from S1d to S3d is not only marked by substantial water loss but also represents a critical phase of material changes. As the number of drying days increases, the moisture content in seeds gradually decreases, which leads to a leveling off of changes in the storage substances, and the seeds progressively enter a dormant state.

### 4.2 The transformation of amino acids and proteins during the drying process of the peanut seeds

During the drying maturation phase of the seeds, the accumulation of storage reserves gradually peaks, and the seeds then undergo a significant process of dehydration. This leads to a drastic reduction in their moisture content to extremely low levels. At this stage, the free amino acids begin their conversion into proteins. This transformation is particularly evident in the amino acids that are crucial for the metabolic conversion of proteins, lipids, and carbohydrates (Amir et al., 2018; Tian et al., 2024; Wang et al., 2024). Glucogenic and ketogenic amino acids, two essential categories, undergo metabolic transformations into glucose and acetyl-CoA, respectively. Acetyl-CoA serves as a fundamental precursor for the biosynthesis of fatty acids (Akram et al., 2011). Although the metabolism of these amino acid categories has been extensively studied in fields, such as hepatic metabolism and epilepsy, they also play vital roles in the conversion of amino acids into fats and carbohydrates in plants (Williamson et al., 1967; Yudkoff et al., 2001).

In this study, we observed significant changes in the patterns of accumulation of various amino acids and their derivatives during the drying of mature peanut kernels. Notably, a variety of glucogenic amino acids, including L-arginine, L-aspartic acid, glutamine, L-glutamic acid, and proline, accumulated significantly in both the early stages (S0d and S1d) and late stages (S7d) of drying. These amino acids may contribute significantly to the accumulation of sugar intermediates, such as glucose-6-phosphate, fructose-6-phosphate, and fructose-1,6-bisphosphate, and glucose observed at S3d and S7d. The high accumulation of ketogenic amino acids at S1d may promote the onset of the accumulation of lipids during this phase. Moreover, arginine, a primary amino acid in peanuts, was consistently identified across all four comparative groups and was found at the highest level in fresh kernels (Korus, 2014). It is also recognized as one of the most nutritionally valuable amino acids owing to its life-extension properties (Arya et al., 2016). Glutamic acid, which increases in content during seed dehydration, reached its maximum level of accumulation at S7d. This is consistent with the accumulation pattern of glutamic acid during drying of rice and wheat (Zhou et al., 2015; Samarah et al., 2025). The upregulation of the expression of glutamate dehydrogenase during the same period may have been related to its catalysis of glutamic acid biosynthesis, thereby promoting the accumulation of glutamic acid during the later stages of drying. Additionally, the content of seed proteins in soybean has been shown to positively correlate with free asparaine (Pandurangan et al., 2012). In this study, the accumulation of L-asparagine varied and ultimately culminated in its maximum accumulation at S7d, which suggested that its levels of protein may have increased during this period. The WGCNA results showed that in the red module highly correlated with 51 amino acids, most genes were involved in the biosynthesis and metabolism of glycogen amino acids and ketogenic amino acids, and were highly expressed in S1d. loc112783205 and loc112736559 respectively encoded aspartate aminotransferase involved in glutamate synthesis, which further demonstrated that the accumulation level of amino acid metabolites was highest in S1d.

In addition, γ-aminobutyric acid plays a crucial role in coping with oxidative stress (Bouche and Fromm, 2004). In our study, γ-aminobutyric acid reached its maximum accumulation level at S3d. This pattern differs from the increase in γ-aminobutyric acid content observed in Arabidopsis seeds after desiccation (Fait et al., 2006). This also confirms that S3d is the primary period during which peanut seeds respond to oxidative stress.

### 4.3 Lipid accumulation and fatty acid biosynthesis during the drying process of peanut seeds

Plant oil primarily originates from plant seeds and is predominantly found in the form of triacylglycerols (TAGs). There are three stages involved in the biosynthesis of oil. First is the production of long-chain fatty acids within plastids; the assembly of these fatty acids with glycerol to form TAGs in the endoplasmic reticulum, and the binding of TAGs with oleosin to produce oil bodies (Hills, 2004). This study highlights the significance of S0d and S1d in the biosynthesis of oil during which related genes were highly expressed. The ratio of oleic acid to linoleic acid, a crucial indicator of the quality of peanuts, was found to be particularly advantageous in peanuts with a high content of oleic acid (López et al., 2001). Our findings showed that stearoyl-ACP desaturase (SAD) catalyzes the conversion of C18:0 ACP toC18:1 ACP. In our study, the upregulation of SAD involved in the biosynthesis of oleic acid from S0d to S1d suggests a potential peak in oleic acid content after one day of drying (López Alonso et al., 2003; Thompson et al., 1991). Conversely, the accumulation of linoleic acid was predominantly observed at S5d and S7d, and it is regulated by both lipoxygenase (LOX) and FAD2. Additionally, the biosynthesis of α-linolenic acid, which is encoded by FAD8, was also upregulated from S0d to S1d. This indicates that the contents of oleic acid may concurrently reach their peaks during the initial drying phase (Sun et al., 2018). This observation was potentially related to the later accumulation of linoleic acid and other contributing factors. Notably, the accumulation of sn-glycero-3-phosphocholine peaked at S7d. As a substrate for phosphocholine synthesis, linoleic acid is metabolized to generate jasmonic acid, thereby influencing seed dormancy. Consequently, the distinctive characteristic of the heightened accumulation of sn-glycero-3-phosphocholine at S7d, which differentiated the dried peanuts from the fresh ones, may be associated with the transition of the seeds into the dormancy phase.

### 4.4 Sugar metabolism and energy provision during the drying process of peanut seeds

As the seeds develop, soluble sugars, such as sucrose, fructose, and glucose, which serve as vital storage substances, provide the essential material for seed germination and early seedling growth. Sugar metabolism not only supplies nutrients and energy for seed growth and development but is also closely linked to the accumulation of storage substances (Rosche et al., 2002; Weichert et al., 2010; Liu et al., 2025). Studies have shown that sucrose is the most abundant sugar in peanuts (Basha, 1992). The accumulation of soluble sugars, including sucrose, glucose, and fructose, occurred significantly at S7d. The biosynthesis of sucrose primarily involves the catalysis of key enzymes, such as SPS, SPP, and SUS (Geigenberger and Stitt, 1993; Lunn and MacRae, 2003). The related genes that promote the accumulation of sucrose were highly expressed at both S5d and S7d. Sucrose is not only used as a raw material for the biosynthesis of other substances, such as starch, oil, and proteins, but its metabolism is also closely associated with various physiological activities of the seeds. From S1d to S3d, glycolysis, as well as starch and sucrose metabolism, were significantly enriched, while the related genes were downregulated at S3d compared to S1d. This suggests that starch may have accumulated abundantly at S0d and S1d. Furthermore, glucose and fructose, the dominant monosaccharide components in peanuts, were observed to rapidly decrease and eventually disappear during the dehydration process of wheat (Lehner et al., 2006). In this study, we found that the accumulation patterns of glucose and fructose during the drying process were similar to that of sucrose, all reaching their peak accumulation levels on the seventh day (S7d). This specific accumulation pattern is likely a unique phenomenon in peanut seed. In contrast, the pattern of accumulation of the soluble sugars exhibited a trend opposite to that of the lipids during seed development, which reflects the preferential utilization of sugars during the biosynthesis of oil (Liu et al., 2024). Our study indicated that glucose was converted into acetyl-CoA through glycolysis and subsequently into fats stored in the seeds. In addition, we utilized the Weighted Gene Co-expression Network Analysis (WGCNA) technique to successfully identify two genes encoding 6-phosphofructokinase (specifically, loc112706323 and loc112722787) and one gene encoding invertase (loc112770733). Notably, the two 6-phosphofructokinase genes play crucial roles as key enzymes in the glycolytic pathway, whereas the invertase gene functions as a key enzyme in the metabolism of starch and sucrose (Plaxton, 1996). The elevated expression levels of these three genes during the initial stages of drying serve as a significant indicator of the ongoing conversion of sugars into oils and starches. Furthermore, the fatty acid biosynthetic pathways were significantly enriched from S1d to S3d. Collectively, these results indicate that the period from S0d to S3d is a crucial stage for the conversion of sugars into fats and starch.

### 4.5 Accumulation of flavonoids during the drying process of peanut seeds

Flavonoids are important secondary metabolites in peanut kernels. They play a significant role not only in regulating the pigmentation of seed coats but also in exhibiting potent antioxidant capabilities (González-Paramás et al., 2019; Peng et al., 2019; Wang et al., 2008). Quercetin has been identified as the major flavonol component in peanut seeds (Hou et al., 2020). This study successfully characterized 112 flavonoid compounds, among which only 15 showed marked alterations in concentration. Intriguingly, the majority of these fluctuating compounds belong to the flavonol class, with quercetin serving as a central flavonoid in peanut seeds. The results revealed that the flavonoids first gradually accumulated from S3d (**Supplementary Figure 6**). FLS catalyzes the conversion of dihydroflavonols to flavonols (Czemmel et al., 2009; Luo et al., 2016). The heightened expression of FLS at S0d may explain the increased accumulation of quercetin observed at S3d. The biosynthesis of plant flavonoids relies on the phenylpropanoid metabolic pathway. The biosynthesis of plant flavonoids relies on the phenylpropane metabolic pathway. In this pathway, PAL acts as the initial rate limiting enzyme, catalyzing the conversion of phenylalanine to cinnamic acid, providing sufficient substrates for subsequent flavonoid synthesis (Dixon and Paiva, 1995). In addition, early upregulation of PAL enhanced phenylpropane metabolic flux, promoting downstream flavonoid synthesis pathways (such as CHS, CHI, FLS gene expression), thereby promoting the maximum accumulation mode of multiple flavonoids in S3d (Ali et al., 2011). CHS, CHI, and F3H catalyze the formation of flavonoid precursors (Premathilake et al., 2020; Wanner et al., 1995., Winkel-Shirley, 2002). In this study, elevated levels of transcription of the PALs, 4CLs, CHSs, and CHIs were observed at S0d and S1d, which could potentially support the significant accumulation of various flavonoids starting from S3d. Notably, among the 15 flavonoid differential metabolites that were screened, rutin had a distinctive pattern of accumulation and peaked at S7d. This pattern may be owing to the increased accumulation of isoquercitrin, a precursor for the biosynthesis of rutin, during S3d and S5d. Furthermore, this pattern of accumulation may serve as a crucial biochemical marker to distinguish between dried and fresh peanut kernels (**Figure 7D**).

### 4.6 Mechanisms of cellular membrane protection and dehydration tolerance during the drying process of peanut seeds

The cellular membrane plays a crucial role in maintaining cellular stability and facilitating normal physiological and biochemical reactions, and it serves as a vital protective barrier for the cells (Li et al., 2023). Lipids, including phospholipids, lysophospholipids, glycerolipids, sterols, and sphingolipids, constitute the structural backbone of the cellular membrane (Xu et al., 2021). Lysophospholipids, intermediates that are formed from phospholipid hydrolysis, occupy a secondary position within the phospholipid bilayer and function as bioactive lipid signaling molecules. In response to low-temperature stress, plants hydrolyze phospholipids and polyunsaturated fatty acids to preserve the fluidity and integrity of their cellular membranes (Tan et al., 2020; Zhang et al., 2023). This hydrolysis significantly enriches the pathways related to linoleic acid metabolism, α-linolenic acid metabolism, and fatty acid biosynthesis during S1d and S3d in peanut seeds. This results in the accumulation of various lysophospholipids and unsaturated fatty acids that maintain membrane integrity and fluidity and prevent damage from cellular dehydration. In coffee beans and Arabidopsis seeds, the content of free fatty acids increases after drying compared to that in fresh seeds (Dussert et al., 2006; Fait et al., 2006). In contrast, peanuts, as a typical oilseed crop, may exhibit more complex metabolic dynamics due to their high content of unsaturated fatty acids and abundant polyphenols. These differences highlight the importance of conducting specific research on oilseed crops. In our study, multiple unsaturated fatty acid and lysophospholipids accumulated significantly at S3d and decreased by S7d, indicating that this stage is critical for peanut seeds to cope with membrane oxidative damage and provide energy for production.

Studies have demonstrated that FAD2 and lipoxygenase (LOX) play roles in maintaining seed membrane fluidity, coping with oxidative damage, and seed dormancy. FAD2 catalyzes the conversion of oleic acid to linoleic acid, influencing the composition of unsaturated fatty acids and thereby regulating lipid fluidity (Okuley et al., 1994). Lipoxygenase (LOX) is a key enzyme that catalyzes the oxidative decomposition of polyunsaturated fatty acids such as linoleic acid and α-linolenic acid, participating in the process of lipid peroxidation. FAD2 is highly expressed in S0d and S1d, increasing the proportion of unsaturated fatty acids to enhance membrane fluidity. Six LOXs (loc112695228, loc112734832, loc112736764, loc112758622, loc112788860, loc112792576) are highly expressed during the early stages of drying (S0d and S1d), potentially experiencing oxidative stress. Lipoxygenases help seeds cope with oxidative damage by regulating lipid oxidation pathways (Porta and Rocha-Sosa, 2002). Four LOXs (loc112714587, loc112733497, loc112791475, loc112783250) are highly expressed during the later stages of drying (S5d and S7d), which may be related to the involvement of signaling molecules such as jasmonic acid produced by lipoxygenases in the induction of seed dormancy, helping seeds maintain dormancy in unfavorable environments. They are then downregulated at S3d to mitigate lipid peroxidation and preserve seed quality (Feussner and Wasternack, 2002).

Soluble sugars plays a crucial role in preventing the transformation of intracellular water into a vitreous state during the dehydration of seeds, thereby protecting the cells from potential damage (Koster, 1991). In normal seeds that undergo mature dehydration, the content of soluble sugars increases (Sun et al., 1994). The ‘water replacement hypothesis’ regarding the mechanism that underlies the formation of seed dehydration tolerance posits that sugars can replace water molecules on the surface of macromolecules, thereby maintaining the stability of the membrane system in a dehydrated state and functioning as effective protectants for this system (Koster and Leopold, 1988). In this study, sucrose and glucose reached their peak accumulation at S7d, which may be associated with the continuous acquisition of dehydration tolerance by seeds during the drying process. Trehalose, a type of oligosaccharide, acts as a primary metabolic inhibitor involved in seed dehydration, with its pattern of accumulation gradually increasing throughout the drying process (Mohanan et al., 2023; Sun et al., 1994). *TPS*s and *TPP*s maintained high levels of expression during S0d and S1d, which correlates with the significant accumulation of trehalose at S7d; this indicates its involvement in the tolerance to seed dehydration and vitrification processes (Hoekstra et al., 2001; Obendorf, 1997).

In addition, various flavonoid metabolites also exhibited a high accumulation pattern during this period, which is attributed to their ability to reduce solute permeability, thereby limiting damage in the early stages of germination (Pourcel et al., 2007). The changes in substances are directly related to the loss of moisture content, thus confirming that the period from S1d to S3d is crucial for peanut drying. During this time, lowering the temperature to reduce the rate of moisture loss in seeds plays a vital role in improving peanut quality.

Drying is the final critical stage in seed development, serving as a bridge between seed maturation and germination. By using multi omics methods to study the dynamic changes of substances in peanuts under natural drying conditions, we aim to elucidate the relationship between gene expression and metabolite accumulation during peanut seed drying, and analyze the dynamic changes and molecular mechanisms of substances. By optimizing the artificial drying conditions, we can improve seed quality and enhance storage stability.

## Data Availability

The raw RNA-seq data generated in the study is available at the SRA database in National Center for Biotechnology Information (NCBI) with the accession number PRJNA1212227 (https://www.ncbi.nlm.nih.gov/bioproject/PRJNA1212227).

## Supplementary material

Supplementary Figure 1 Volcano plot showing the number of up-regulated or down-regulated different expression genes in different comparison groups.

Supplementary Figure 2 GO enrichment circle chart.

Supplementary Figure 3 Expression heatmaps of genes in the red and cyan modules during the drying process of peanut seeds.

Supplementary Figure 4 KEGG pathway of red and cyan modules.

Supplementary Figure 5 KEGG enrichment bubble plot of 10078 differentially expressed genes.

Supplementary Figure 6 Cluster heatmap of amino acids and their derivatives, lipids, Carbohydrates and its derivatives heatmap, and Flavonoids metabolites.
