# Supplementary material for: Integrative analysis of transcriptome and metabolome reveals molecular mechanisms of dynamic change of storage substances during dehydration and drying process in peanuts (Arachis hypogaea L.)
Source: Front Plant Sci. 2025 Apr 16;16:1567059. doi: 10.3389/fpls.2025.1567059 (PMC12042706; doi:10.3389/fpls.2025.1567059)
Supplement: Supplementary Figure 1 — Volcano plot showing the number of up-regulated or down-regulated different expression genes in different comparison groups. [file DataSheet1.pdf]

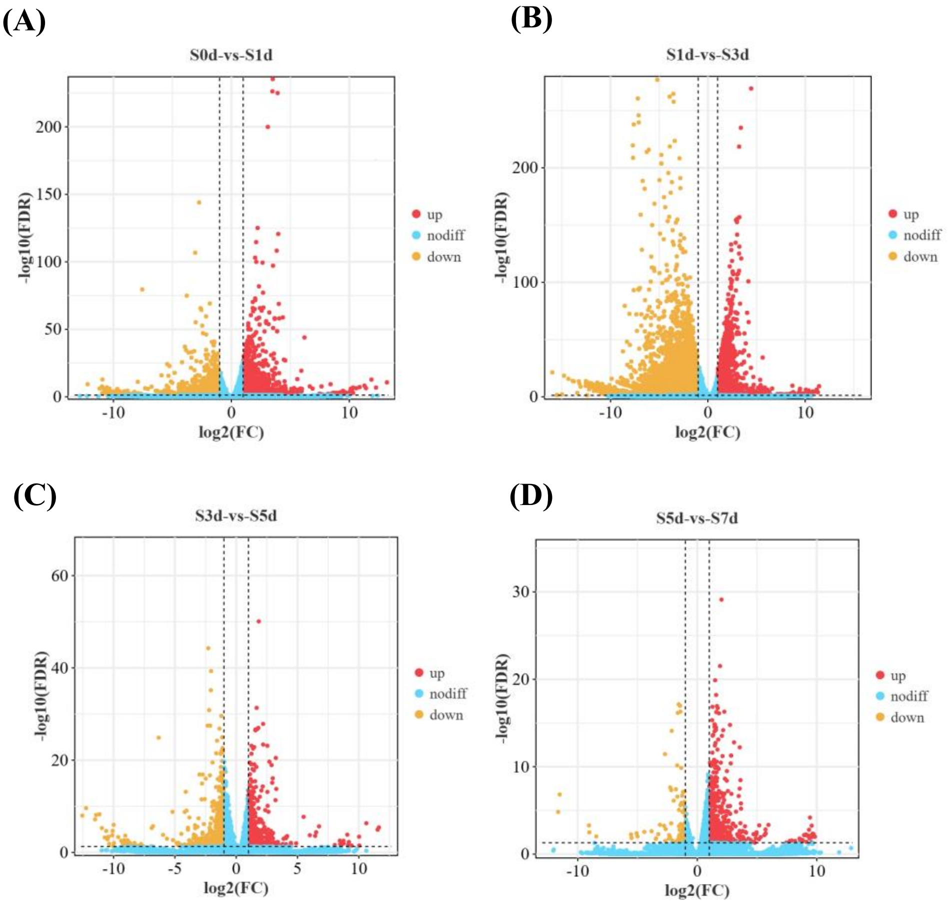

Supplementary Figure 1 Volcano plot showing the number of up-regulated or down-regulated differential genes in different comparison groups. **(A)** S0d-vs-S1d differential genes volcano plot; **(B)** S1d-vs-S3d differential genes volcano plot; **(C)** S3d-vs-S5d differential genes volcano plot; **(D)** S5d-vs-S7d differential genes volcano plot. The X-axis represents log2(Fold Change) and the Y-axis represents -log10(*P*-value). The two vertical dashed lines represent the threshold of expressing the multiple of difference and the horizontal dashed lines represent the threshold of significance level. Different colors of red, orange and blue represent up-regulated, down-regulated and no significant difference of DEGs, respectively.

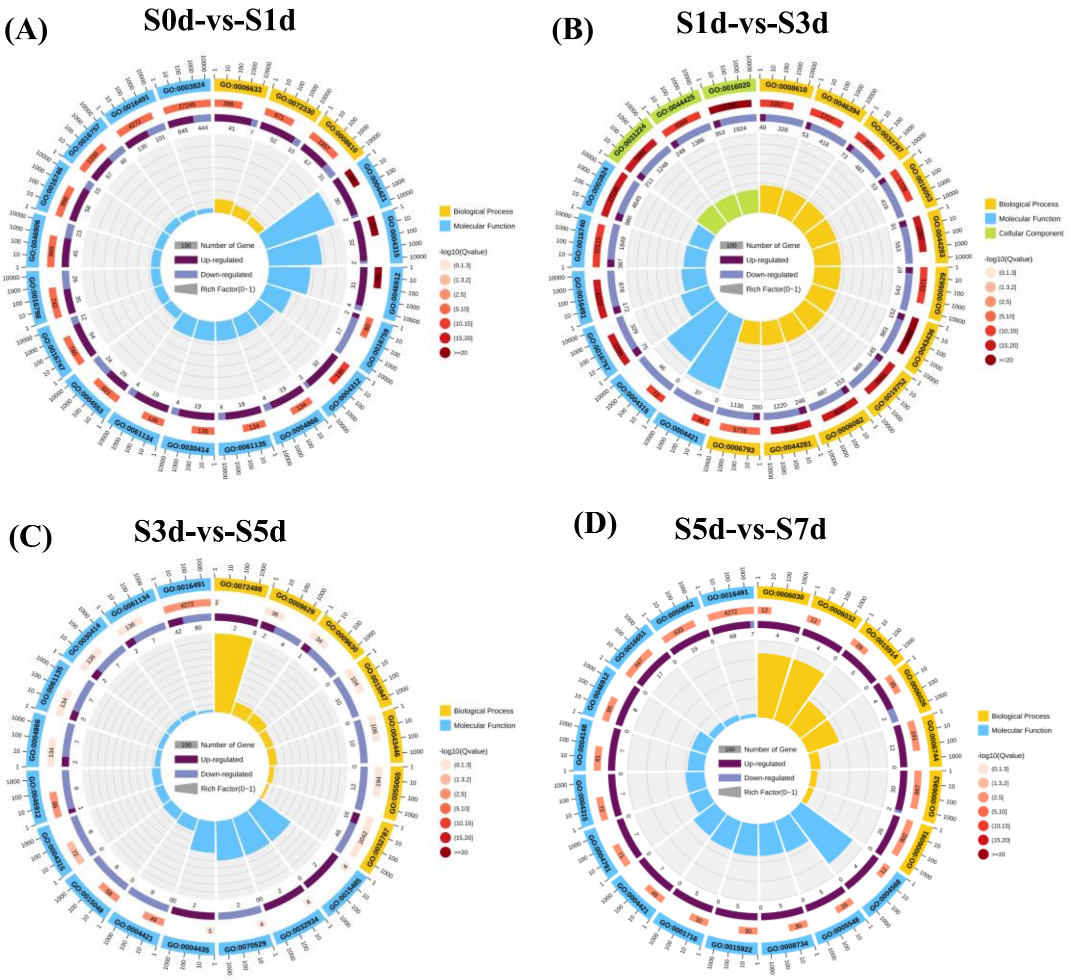

Supplementary Figure 2 GO enrichment circle chart. **(A)** S0d-vs-S1d differential genes GO enrichment circle chart; **(B)** S1d-vs-S3d differential genes GO enrichment circle chart; **(C)** S3d-vs-S5d differential genes GO enrichment circle chart; **(C)** S5d-vs-S7d differential genes GO enrichment circle chart. (First circle: top 20 enriched GOterms, outside the circle is the coordinate scale of the number of differentially expressed genes. Different colors represent different ontologies; Second circle: the number and Q value of the GOterm in the differentially expressed gene background. The more differentially expressed gene backgrounds there are, the longer the bar, and the smaller the Q value, the redder the color.); Third circle: Bar chart of the proportion of upregulated and downregulated differentially expressed genes, with dark purple representing the proportion of upregulated differentially expressed genes and light purple representing the proportion of downregulated differentially expressed genes; The specific numerical values are displayed below; Fourth circle: RichFactor values for each GOterm (the number of differentially expressed genes in that GOterm divided by the total number in that GOterm), background gridlines, with each grid representing 0.1).

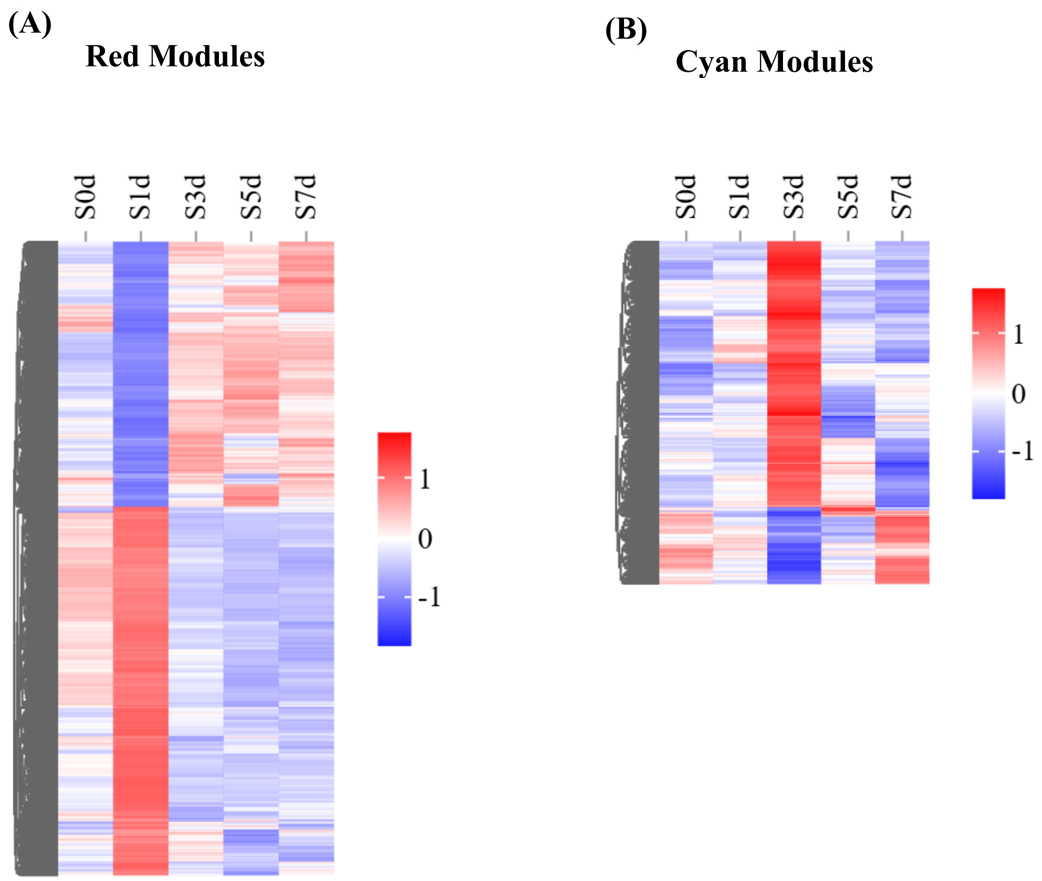

Supplementary Figure 3 Expression heatmaps of genes in the red and cyan modules during the drying process of peanut seeds. **(A)** Gene expression heatmap in red module; **(B)** Gene expression heatmap in cyan module

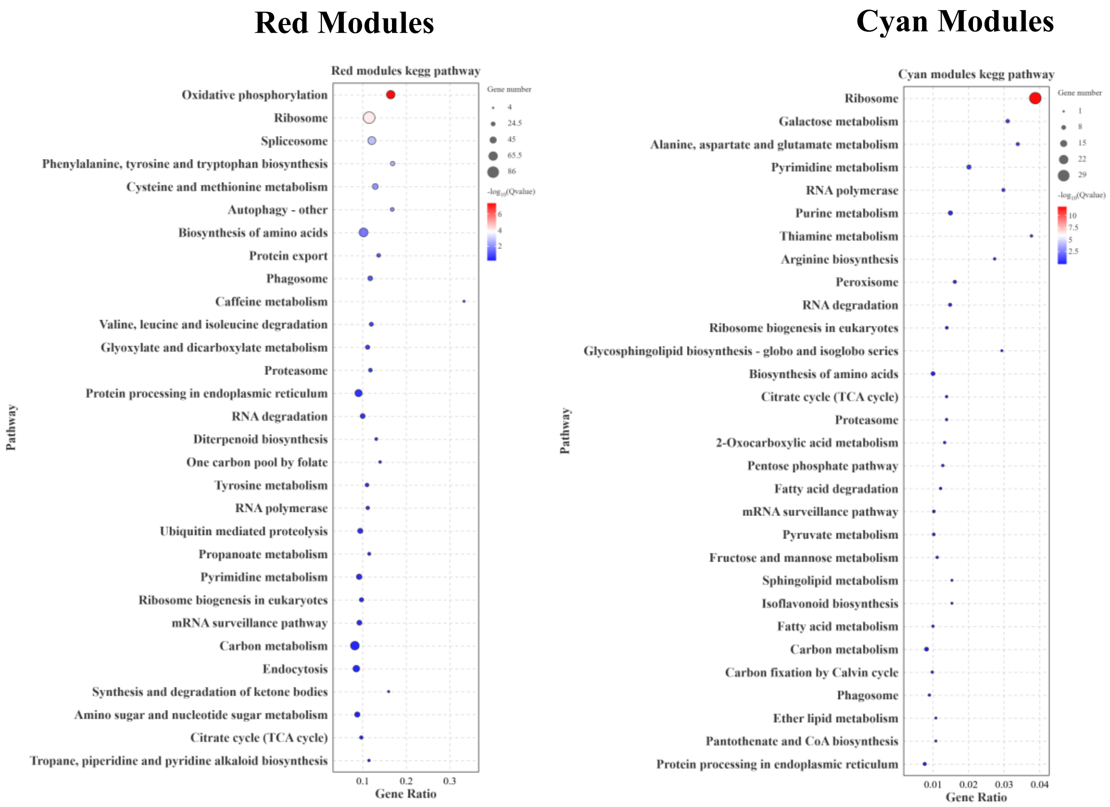

Supplementary Figure 4 KEGG pathway of red and cyan modules. **(A)** KEGG pathway of red module; **(B)** KEGG pathway of cyan module. Use the top 30 pathways with the lowest P-value to plot, with the y-axis representing the pathway and the x-axis representing the enrichment factor. The size of the bubbles indicates the number of target genes enriched in the pathway, and the color of the bubbles indicates the significance of enrichment in the pathway. The larger the value, the more significant the enrichment.

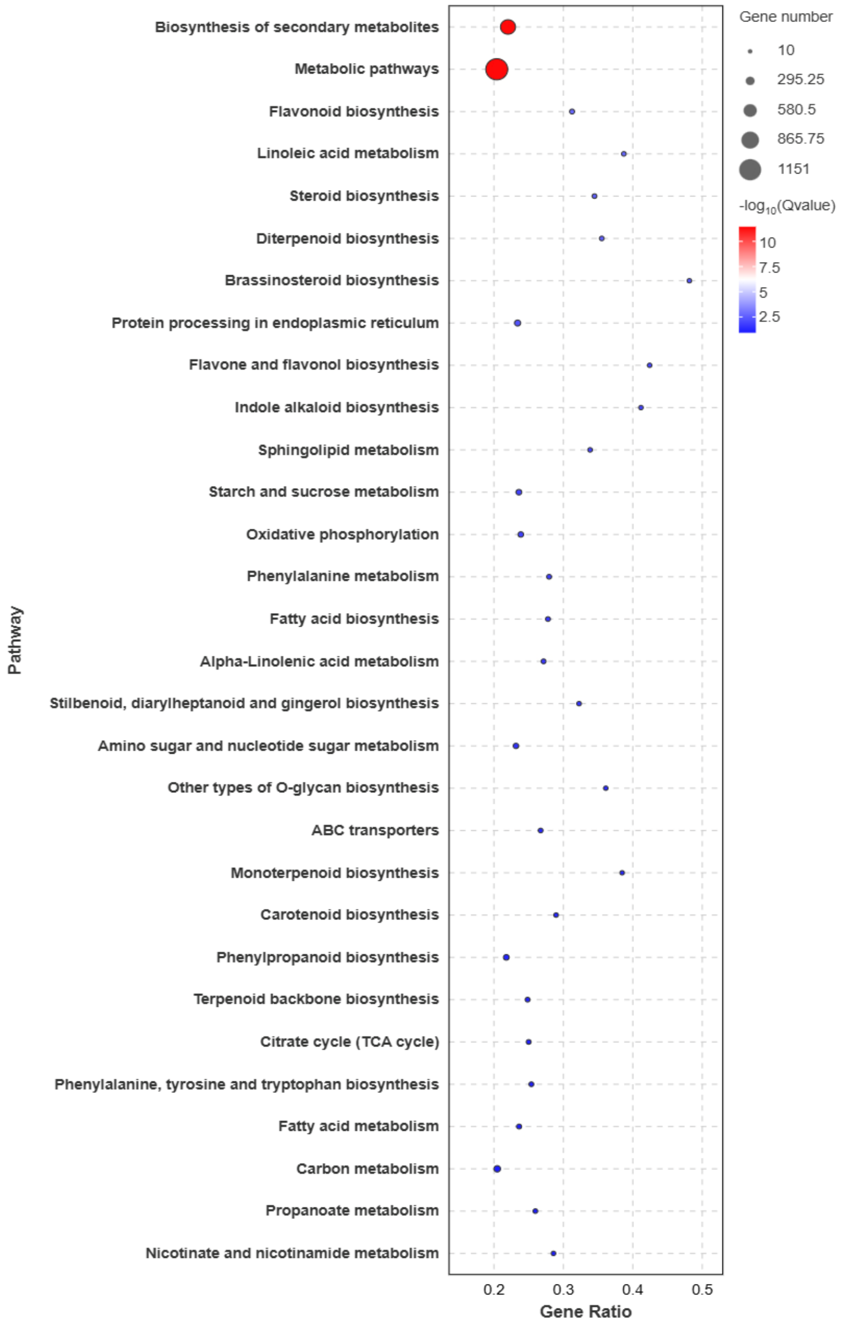

Supplementary Figure 5 KEGG enrichment bubble plot of 10078 differentially expressed genes. Use the top 30 pathways with the lowest P-value to plot, with the y-axis representing the pathway and the x-axis representing the enrichment factor. The size of the bubbles indicates the number of target genes enriched in the pathway, and the color of the bubbles indicates the significance of enrichment in the pathway. The larger the value, the more significant the enrichment.

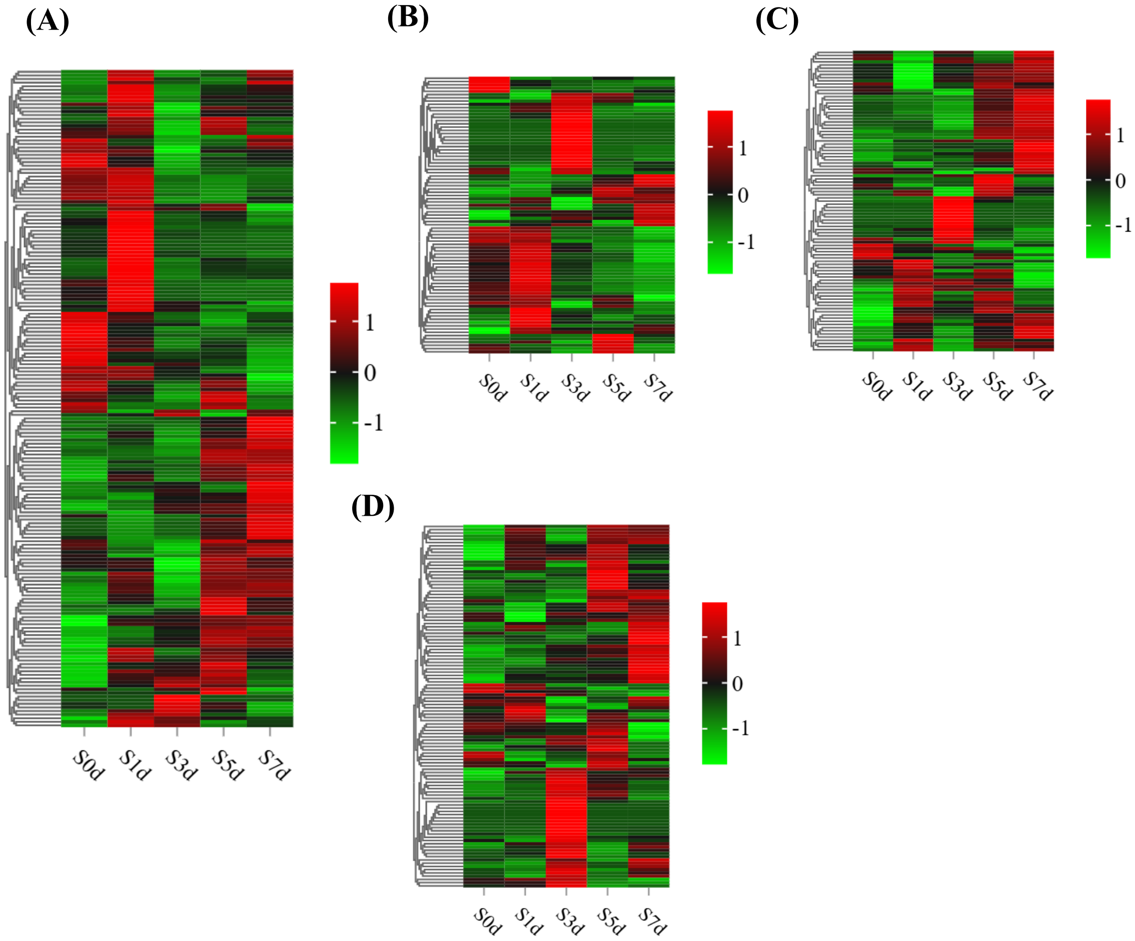

Supplementary Figure 6 Cluster heatmap of amino acids and its derivatives, lipids, Carbohydrates and its derivatives heatmap, and Flavonoids metabolites. **(A)** The heatmap of Amino Acids and their Derivatives; **(B)** The heatmap of Lipids; **(C)** The heatmap of Carbohydrates and its Derivatives; **(D)** The heatmap of Flavonoids
